# Two new sulfur-containing pyridine alkaloids from the co-culture of *Diaporthe* fungi

**DOI:** 10.3389/fmicb.2026.1963522

**Published:** 2026-10-07

**Authors:** Ji-Xin Li, Yu-Shan Gong, Meng-Xue Lu, Hong-Jie Shen, Hao-Xian Lu, Xiu-Juan Zhao, Tian-Peng Yin, Siwen Yuan

**Affiliations:** 1 School of Bioengineering, Zunyi Medical University, Zhuhai, Guangdong, China; 2 Guizhou Key Laboratory of Integrated Traditional Chinese and Western Medicine, Zunyi Medical University, Zhuhai, China; 3 Southern Marine Science and Engineering Guangdong Laboratory (Zhuhai), Zhuhai, China

**Keywords:** antifungal activity, co-culture, genome mining, natural products, sulfur-containing pyridine alkaloids

## Abstract

The untapped metabolome of filamentous fungi represents a profound reservoir for therapeutic lead discovery. Although genomic mining confirms an abundance of cryptic biosynthetic gene clusters (BGCs), axenic laboratory culture frequently lacks the ecological stimuli required to trigger their expression, inevitably leading to the redundant isolation of known metabolites. To circumvent this biosynthetic silencing and unlock hidden chemical diversity, strategies such as heterologous expression, the one-strain-many-compounds (OSMAC), epigenetic modulation, and microbial co-cultivation have been increasingly deployed. Herein, we harnessed an interspecies co-cultivation strategy to systematically investigate the biogenetic potential of mangrove-associated fungi belonging to the genus *Diaporthe*. Four fungal strains (*Phomopsis phyllanthicola* ZMU-32-2-1, *Diaporthe kyushuensis* ZMU-48-1, *Diaporthe phaseolorum* ZMU-49-3, and *Diaporthe* sp. ZMU-11) were initially isolated and purified. Subsequent co-cultivation screening revealed that strains ZMU-48-1, ZMU-32-2-1, and ZMU-11 showed altered metabolic profiles, with the appearance of novel chromatographic peaks that were not observed in their respective monocultures. Scaled-up co-fermentation and subsequent chromatographic purification led to the isolation of five structurally diverse compounds. Notably, this included two unprecedented sulfur-containing pyridine alkaloids, designated as diaridine A (1) and B (2), alongside three previously described secondary metabolites. The isolated compounds were evaluated for their antifungal properties; however, none displayed significant inhibitory activity at a concentration of 200 μg/mL. Despite the absence of potent antifungal efficacy, these findings highlight the power of microbial crosstalk in activating the cryptic chemical space of *Diaporthe* species, reaffirming their potential as a robust resource for novel drug discovery.

## 1 Introduction

Fungal natural products are recognized as one of the most important sources of structurally diverse and biologically active small molecules for pharmaceutical and agrochemical development, exemplified by milestone compounds such as penicillin, griseofulvin, and echinocandin-derived antifungal agents (Demain and Sanchez, 2009; Keller, 2019). Owing to their remarkable chemical diversity and broad spectrum of biological activities, fungal secondary metabolites have long served as valuable leads for the discovery of antibacterial, antifungal, antiviral, anticancer, and anti-inflammatory agents (Netzker et al., 2015; Keller, 2019; Pinar and Rodríguez-Couto, 2024). With the rapid development of whole-genome sequencing and genome mining tools, it has become increasingly evident that the biosynthetic potential encoded in fungal genomes far exceeds the number of metabolites that can be detected under conventional laboratory conditions (Netzker et al., 2015). Large-scale genomic analyses have shown that only a very small fraction of predicted fungal biosynthetic gene clusters (BGCs) can currently be correlated with known natural products, indicating that the majority of fungal metabolic potential remains unexplored (Robey et al., 2021; Zhang et al., 2024). However, many of these BGCs are silent or only weakly expressed in axenic culture, largely because routine cultivation conditions fail to reproduce the complex biotic and abiotic stimuli present in natural ecological niches (Netzker et al., 2015). Consequently, the activation of cryptic biosynthetic pathways has become a key objective in fungal natural product discovery.

To overcome this bottleneck, several complementary approaches have been developed to stimulate the expression of silent fungal BGCs, including heterologous expression, the one-strain-many-compounds (OSMAC) strategy, epigenetic modulation, transcriptional engineering, and microbial co-cultivation (Knowles et al., 2022; Xu et al., 2023; Zakariyah et al., 2024; Wang and Mao, 2025). Among these, co-cultivation has attracted particular attention because it provides an experimentally simple yet ecologically meaningful means of mimicking interspecies interactions, thereby inducing metabolic reprogramming and awakening otherwise cryptic secondary metabolism (Yu et al., 2021). The underlying mechanisms may involve direct physical contact, diffusible chemical signals, nutrient competition, or stress-triggered regulatory responses, all of which can alter fungal biosynthetic outputs (Schroeckh et al., 2009). The success of this strategy has been demonstrated by several representative examples. The potent antibiotic pestalone was discovered from the co-culture of the marine fungus *Pestalotia* sp. with a marine bacterium, representing a classic case of chemically induced metabolite production (Cueto et al., 2001). Likewise, co-cultivation of *Libertella* sp. with a marine bacterium yielded libertellenones A-D, a group of cytotoxic diterpenoids absent in monoculture (Oh et al., 2005). In fungal-fungal interaction systems, citrifelins A and B were isolated from the co-culture of *Penicillium citrinum* and *Beauveria felina*, further illustrating the capacity of co-culture to generate structurally unusual metabolites (Meng et al., 2015). These studies collectively demonstrate that co-cultivation is an effective strategy for accessing hidden fungal chemistry and expanding the diversity of discoverable natural products (Knowles et al., 2022).

Species of the genus *Diaporthe* (anamorph: *Phomopsis*) are widely distributed as endophytes, saprobes, and plant pathogens in a broad range of terrestrial and marine-associated environments, and are increasingly recognized as prolific producers of bioactive secondary metabolites (Xu et al., 2021; Wei et al., 2023). Previous chemical investigations have revealed that *Diaporthe* species are capable of producing a wide variety of natural products, including polyketides, pyrones, alkaloids, terpenoids, steroids, fatty acids, benzophenones, and other structurally diverse metabolites (Xu et al., 2021). Many of these compounds exhibit cytotoxic, antibacterial, antifungal, antiviral, anti-inflammatory, antioxidant, and phytotoxic activities, highlighting the potential of this genus as a reservoir of pharmacologically and agriculturally relevant molecules (Xu et al., 2021). Recent reviews have summarized hundreds of metabolites from *Diaporthe* and *Phomopsis*, underscoring both their extensive chemical diversity and their continued importance in natural product discovery (Xu et al., 2021). Moreover, genome-based analyses have suggested that *Diaporthe* is among the fungal genera with considerable biosynthetic potential. In our previous studies, fungal isolation, chemical investigation, and whole-genome sequencing revealed that *Diaporthe* strains generally harbor around or more than one hundred putative BGCs, strongly suggesting that they encode a much broader secondary metabolome than has been experimentally characterized to date (Zheng et al., 2025). Nevertheless, only a limited fraction of these BGCs appears to be expressed under routine cultivation conditions, making the activation of silent gene clusters particularly important for accessing the hidden chemical space of *Diaporthe* (Wang and Mao, 2025).

In the present work, four *Diaporthe*-related fungal strains with different ecological origins were investigated through interspecific co-cultivation. Among them, *P. phyllanthicola* ZMU-32-2-1, *D. phaseolorum* ZMU-49-3, and *Diaporthe* sp. ZMU-11 were isolated from mangrove leaves, whereas *D. kyushuensis* ZMU-48-1 was obtained from decayed leaves of *Acacia confusa* Merr. The inclusion of strains from distinct yet plant-associated ecological sources provided an opportunity to probe whether interspecific interaction could stimulate otherwise silent metabolic pathways and enrich their chemical outputs. While most co-culture studies have used two strains, we attempted a tripartite co-culture system to explore whether a three-strain combination could further promote interspecific interactions and differential metabolite production. Through co-culture screening and comparative HPLC profiling, we identified a culture combination that displayed an altered metabolic profile, including the appearance of additional chromatographic peaks relative to the corresponding monocultures. Subsequent scale-up fermentation and chromatographic purification led to the isolation of two new sulfur-containing pyridine alkaloids, diaridine A (1) and diaridine B (2), together with three known metabolites (Figure 1). In addition, the isolated compounds were evaluated for antifungal activity against a panel of phytopathogenic fungi. These findings further support co-cultivation as a practical and effective strategy for activating cryptic fungal secondary metabolism and for uncovering structurally novel natural products from *Diaporthe* species.

**FIGURE 1:**
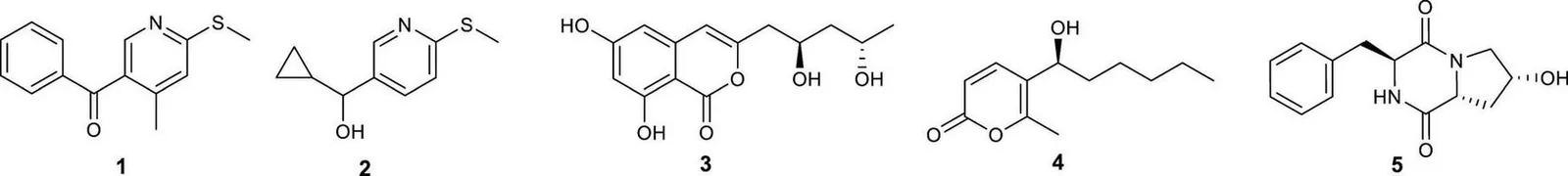
The structures of secondary metabolites 1–5.

## 2 Materials and methods

### 2.1 General experimental procedures

Optical rotations were determined using an Anton Paar MCP 200 polarimeter. Infrared (IR) absorption spectra were recorded on an Agilent Cary 630 Fourier transform infrared spectrometer. 1D and 2D nuclear magnetic resonance (NMR) spectra were acquired on a Bruker AVANCE III 600 MHz spectrometer equipped with a 5 mm CPP BBO cryoprobe (CPP BBO600S3 BB-H&F-D-05 Z XT). Chemical shifts were referenced to the residual solvent signals of methanol-*d*_4_ and DMSO-*d*_6_, which were supplied by Cambridge Isotope Laboratories, Inc. (USA). High-resolution electrospray ionization mass spectrometry (HRESIMS) measurements were conducted utilizing a Thermo Orbitrap Fusion Lumos mass spectrometer. Preparative high-performance liquid chromatography (HPLC) purifications were carried out on a Waters system consisting of a 1525 binary pump and a 2998 photodiode array (PDA) detector. The chromatographic separations were achieved using several columns: an Xtimate C18 (10 × 250 mm, 5 μm, Welch Materials), an Ultimate XB-Phenyl (10 × 250 mm, 5 μm, Welch Materials, USA), and an ACE C18 (4.6 × 250 mm, 5 μm, Advanced Chromatography Technology). The eluent was continuously monitored at multiple UV wavelengths (220, 243, 271, and 296 nm). Column chromatography (CC) was performed over silica gel (300–400 mesh), and thin-layer chromatography (TLC) analyses were conducted on precoated silica gel GF254 plates, both of which were sourced from Qingdao Haiyang Chemical Co., Ltd., (China). TLC spots were detected under UV illumination and subsequently visualized by heating after spraying with a vanillin-sulfuric acid reagent. HPLC-grade acetonitrile and methanol were obtained from Tianjin Concord Technology (China). All other analytical-grade organic solvents used for extraction and column chromatography (including methanol, ethanol, dichloromethane, ethyl acetate, and petroleum ether) were procured from Tianjin Damao Chemical Reagent Co., Ltd., (China).

### 2.2 Fungal material and strain identification

Detailed information regarding the isolation, identification, and comprehensive genomic characteristics of the fungal strain *D. kyushuensis* ZMU-48-1 has been extensively described in our previous study (Zheng et al., 2025) and is therefore not reiterated in this paper. The fungus *P. phyllanthicola* ZMU-32-2-1, *D. phaseolorum* ZMU-49-3, and *Diaporthe* sp. ZMU-11 were isolated from fresh mangrove leaves collected from the intertidal zone of Qi’ao Island (Xiangzhou District, Zhuhai, Guangdong Province, China) in October 2023. The fungal strains used in the antifungal assay were obtained from the China General Microbiological Culture Collection Centre (CGMCC).

Taxonomic identification of the fungal isolates was initially assessed using comprehensive bioinformatic analyses based on the internal transcribed spacer (ITS) region, and definitive molecular identification was achieved through targeted phylogenetic analysis. The ITS sequence extracted from the assembled genome was queried against the ITS subdatabase of the NCBI NT database using BLAST, with a sequence identity threshold of >95%. The 30 most closely related homologous sequences with the highest identity scores were then retrieved. Multiple sequence alignment was performed using MAFFT, followed by phylogenetic tree construction with FastTree, IQ-TREE, or RAxML to determine the precise evolutionary placement of the isolate.

### 2.3 Fungal co-cultivation and HPLC analysis

#### 2.3.1 Plate inoculation methods

Photodiode array medium was prepared by dissolving 10.8 g agar (15 g/L) and 14.4 g Potato dextrose broth (PDB; 24 g/L) in 600 mL ultrapure water. The medium was sterilized by autoclaving at 121 °C for 20 min, cooled to approximately 50 °C, and aseptically dispensed into 15 Petri dishes under a laminar flow hood. Four fungal strains (*P. phyllanthicola* ZMU-32-2-1, *D. kyushuensis* ZMU-48-1, *D. phaseolorum* ZMU-49-3, and *Diaporthe.* sp. ZMU-11) were maintained on PDA plates. Each strain was subcultured for three successive generations by transferring mycelial agar plugs to the center of fresh plates. Plates were labeled on the underside, sealed and incubated at 30 °C for 5 days.

Photodiode array medium (200 mL) was prepared according to the same formulation (agar 15 g/L; PDB 24 g/L), sterilized by autoclaving, cooled, and poured into 11 Petri dishes under aseptic conditions. Individual strains were centrally inoculated onto separate plates, labeled on the reverse side, and incubated at 30°C. Colony morphology was documented photographically at 72-h intervals throughout the incubation period. Since most previous fungal co-culture studies have been restricted to two strains, tripartite (three-strain) co-culture systems were designed to explore whether the involvement of a third strain could further promote interspecific interactions and induce the production of differential metabolites. ZMU-11 was selected as the target strain, and candidate tripartite combinations were designed to contain ZMU-11 together with two of the other three strains (ZMU-32-2-1, ZMU-48-1, and ZMU-49-3), so that the antagonistic effect of the accompanying strains on ZMU-11 could be evaluated. To assess interspecific interactions, the following three tripartite combinations–(ZMU-32-2-1, ZMU-49-3, ZMU-11), (ZMU-32-2-1, ZMU-48-1, ZMU-11), (ZMU-49-3, ZMU-48-1, ZMU-11)–were inoculated onto PDA plates in a triangular configuration, with each isolate positioned at an equal distance from the others. Plates were incubated at 30 °C and imaged every 7 days to monitor colony development and interaction zones.

#### 2.3.2 Liquid co-culture methodology and HPLC analysis

Potato dextrose broth (PDB; 24 g/L) was prepared by dissolving 9.6 g PDB powder in 400 mL ultrapure water. The medium was sterilized by autoclaving at 121 °C for 20 min, cooled to approximately 50°C, and aseptically dispensed in 40 mL aliquots into eight 50-mL centrifuge tubes. Based on the three tripartite combinations identified during plate co-culture screening, namely (ZMU-32-2-1, ZMU-49-3, and ZMU-11), ZMU-32-2-1, ZMU-48-1, and ZMU-11), and (ZMU-49-3, ZMU-48-1, and ZMU-11), mycelial plugs (∼1 cm × 1 cm) were harvested from the actively growing margins of 5-day-old colonies of each strain and inoculated into PDB to establish mixed seed cultures. The seed cultures were incubated on a rotary shaker at 150 rpm and 30 °C for 48 h. The resulting mixed seed culture (40 mL) was then transferred into a 500-mL Erlenmeyer flask containing 200 mL of fresh PDB for scale-up fermentation (Supplementary Figure 1). Cultures were incubated on a rotary shaker at 220 rpm and 30 °C for 14 days. Monoculture controls of all four individual strains were established in parallel under identical conditions. After fermentation, the cultures were extracted with methanol (50 mL) and partitioned with ethyl acetate (250 mL) to afford crude extracts. HPLC analysis was performed on an ACE C18 column (4.6 × 250 mm, 5 μm; Advanced Chromatography Technology) at 254 nm with a flow rate of 3 mL/min. The mobile phase consisted of acetonitrile and water under the following gradient conditions: 10%–50% acetonitrile (0–15 min), 50%–100% acetonitrile (15–30 min), 100% acetonitrile (30–35 min), 100%–10% acetonitrile (35–37 min), and 10% acetonitrile (37–40 min). The optimal co-culture combination was selected by integrating the HPLC profiles with the results of the plate co-culture assay. All of the above experiments were performed in duplicate.

Following the procedure described above, the optimal tripartite combination consisting of ZMU-32-2-1, ZMU-48-1, and ZMU-11 was inoculated into PDB to generate a mixed seed culture. After 48 h of incubation, 40 mL of the mixed seed culture was inoculated into each of twenty 500-mL Erlenmeyer flasks containing 200 mL of fresh PDB. All flasks were incubated on a rotary shaker at 220 rpm and 30 °C for 14 days. Monoculture controls of all four individual strains were established in parallel under the same conditions.

### 2.4 Genome sequencing, assembly, and annotation

Genomic DNA extraction and sequencing library preparation for ZMU-32-2-1 and ZMU-11 were performed according to the identical protocols previously described for ZMU-48-1. Detailed methodologies for the DNA extraction, library construction, as well as the complete genome assembly and annotation pipeline for strain ZMU-48-1, have been published previously (Zheng et al., 2025).

For the genome assembly and annotation of strains ZMU-32-2-1 and ZMU-11, the raw sequencing data underwent rigorous quality control. For strain ZMU-32-2-1, fastp was utilized to trim adapter sequences and filter out low-quality bases. Conversely, data for strain ZMU-11 was evaluated using FastQC and processed via Trimmomatic. For both pipelines, reads shorter than 35 bp and bases with a quality score below 20 (*Q* < 20) were removed to yield high-quality clean reads. Contamination assessment was subsequently performed by aligning 10,000 randomly sampled reads against the NCBI NT database. *De novo* genome assembly for both strains was conducted using the SPAdes algorithm, followed by GapFiller to close internal assembly gaps. To address base errors and small insertion/deletion events, sequence correction was applied using Pilon for strain ZMU-32-2-1 and PrInSeS-G for strain ZMU-11 (Bankevich et al., 2012; Boetzer and Pirovano, 2012; Koren et al., 2017). For gene prediction, coding sequences (CDS) were identified using glimmer for ZMU-32-2-1 and GeneMark for ZMU-11 (Delcher et al., 1999; Majoros et al., 2004). Non-coding RNAs were detected using RNAmmer for rRNAs in both strains, alongside aragorn (for ZMU-32-2-1) or tRNAscan-SE (for ZMU-11) for tRNA identification (Tegenfeldt et al., 2025). Repeat sequences in both genomes were *de novo* predicted and classified using RepeatModeler and RepeatMasker. Functional annotation was achieved by comparing the predicted protein sequences against multiple comprehensive databases using NCBI BLAST+, including NR, KOG, CDD, and PFAM. KEGG pathway annotations were systematically assigned using the KAAS server. Additionally, for strain ZMU-32-2-1, carbohydrate-active enzymes were annotated using HMMER3 against the CAZy database, and virulence factors alongside antibiotic resistance genes were identified using the VFDB and CARD databases, respectively. Comparative genomic analyses for ZMU-32-2-1, including pan-genome profiling via Orthofinder2 and gene family evolution assessment using CAFE5, were also conducted to gain deeper insights into its genetic characteristics (Emms and Kelly, 2019).

To systematically evaluate their biosynthetic potential, the genome assemblies and corresponding GFF annotation files of strains ZMU-32-2-1, ZMU-11, and ZMU-48-1 were analyzed using antiSMASH version 7.0 (Blin et al., 2019). This comprehensive platform facilitated the precise identification, classification, and comparative profiling of biosynthetic gene clusters (BGCs), providing detailed insights into the diverse secondary metabolome encoded by each fungal strain.

### 2.5 Isolation and identification of compounds

The liquid cultures were extracted three times with 2 L of ethyl acetate (EtOAc), while the mycelia were sonicated three times in 500 mL of methanol (MeOH). The combined extracts were then concentrated under reduced pressure to afford the total crude extract. The crude extracts were separated into seven fractions (Fr.A1–Fr. A7) with a gradient silica gel CC (PE–EA, 100% to 0). Fr.A2 was further separated via preparative HPLC using an acetonitrile-water system (46.7% acetonitrile) on an Ultimate XB-Phenyl column (10 × 250 mm, 5 μm; Welch Materials) to obtain compounds 1 (4.4 mg, t_*R*_ = 9.298 min). Fr.A4 was further separated via preparative HPLC using an acetonitrile-water system (27% acetonitrile) on an ACE C18 (4.6 × 250 mm, 5 μm, Advanced Chromatography Technology) compounds 2 (2.75 mg, t_*R*_ = 13.481 min). Fr.A5 was further separated *via* preparative HPLC using an acetonitrile-water system (20% acetonitrile) on an Ultimate XB-Phenyl column (10 × 250 mm, 5 μm; Welch Materials) to obtain compounds 3 (2.1 mg, t_*R*_ = 24.293 min). Fr.A3 was further separated *via* preparative HPLC using an acetonitrile-water system (60% acetonitrile) on an Ultimate XB-Phenyl column (10 × 250 mm, 5 μm; Welch Materials) to obtain compounds 4 (1.9 mg, t_*R*_ = 7.441 min). Fr.A6 was further separated via preparative HPLC using an acetonitrile-water system (20% acetonitrile) on an Ultimate XB-Phenyl column (10 × 250 mm, 5 μm; Welch Materials) to obtain compounds 5 (1.56 mg, t_*R*_ = 11.982 min).

Diaridine A (1): white amorphous powder; IR (KBr, cm^–1^): 3419, 3389, 2926, 2851, 1656, 1581, 1534, 1469, 1317, 1280, 1241, 1155, 1085. The ^1^H and ^13^C NMR spectral data are shown in Table 1. HR-ESI-MS *m/z* 244.0778 [M+H]^+^ (calcd for C_14_H_14_NOS^+^, 244.0791).

**TABLE 1:** ^1^H (600 MHz) and ^13^C NMR (150 MHz) data of compounds 1 and 2 in CD_3_OD.

Nos.	1		2	
	δ_H_ (*J* in Hz)	δ_C_	δ_H_ (*J* in Hz)	δ_C_
2	8.34 (1H, s)	149.1 d	8.39 (1H, d, 2.2)	146.7 d
3		130.0 s	/	136.0 s
4		146.6 s	7.71 (1H, dd, 8.3,2.2)	134.5 d
5	7.35 (1H, s)	122.6 d	7.25 (1H, d, 8.3) 3.95 (1H, d, 8.3)	120.2 d
6		162.2 s		159.1 s
7		195.5 s		74.9 d
8	2.26 (3H, s)	19.2 q	2.53 (3H, s)	12.1 q
9	2.56 (3H, s)	12.7 q		
1’		137.3 s	1.13 (1H, m)	18.0 d
2’/6’	7.74 (2H, d, 7.7)	129.7 d	0.63 (1H, m), 0.51 (1H, m)	2.8 t
3’/5’	7.56 (2H, dd, 7.7, 7.4)	128.9 d	0.51 (1H, m), 0.34 (1H, m)	1.4 t
4’	7.70 (1H, t, 7.4)	133.7 d		

Diaridine B (2): white amorphous powder; IR (KBr, cm^–1^): 3441, 3005, 2924, 1593, 1022, 823. The ^1^H and ^13^C NMR spectral data are shown in Table 1. HR-ESI-MS *m/z* 196.0789 [M+H]^+^ (calcd for C_10_H_14_NOS^+^, 196.0791).

### 2.6 Antifungal activity

All compounds were screened for their antifungal properties against a panel of nine phytopathogens, including *F. graminearum*, *B. dothidea*, *F. oxysporum* Schltdl., *C. gloeosporioides*, *V. mali* Miyabe and G. Yamada, *C. musae*, *A. alternata*, *C. agenarium*, and *B. sorokiniana*. The minimum inhibitory concentration (MIC) was determined by a serial macro-dilution assay (Bhat et al., 2011). The fungal strains used in the antifungal assay were obtained from the China General Microbiological Culture Collection Centre (CGMCC). Test compounds were first dissolved in dimethyl sulfoxide (DMSO) and subsequently serially diluted in potato dextrose broth (PDB), with an initial concentration of 200 μg/mL. The final concentration of DMSO in the assay system was maintained at 2% (v/v). Standard inocula of the tested fungi were prepared at a density of approximately 1 × 10^6^ spores/mL. For each assay, 0.2 mL of the test solution was added to 0.2 mL of PDB in a series of microtubes, followed by serial dilution. Then, 0.2 mL of the fungal inoculum was added to each tube, and the mixtures were incubated at 30 °C for 24 h. All assays were performed in triplicate. The MIC was defined as the lowest concentration of the test compound that completely inhibited visible fungal growth after incubation. DMSO and carbendazim were used as the negative and positive controls, respectively.

## 3 Results and discussion

### 3.1 Identification of strains ZMU-32-2-1, ZMU-11 and ZMU-49-3

The taxonomic affiliations of the two fungal strains were evaluated based on their respective genomic sequencing data. For strain ZMU-32-2-1, identification was performed using ITS sequence, which was queried against the NCBI nucleotide database (ITS subdatabase) using the BLAST algorithm with an identity threshold set to >95%. The sequence alignment revealed that the closest related strains were *Phomopsis phyllanthicola* (accession no. EF488374) sharing 99.59% identity, *Phomopsis liquidambari* (FJ478124 and HQ328002) sharing 99.39% and 98.61% identities, respectively, and *Phomopsis loropetali* (AY601917) sharing 98.65% identity. A phylogenetic tree was subsequently constructed using multiple sequence alignment to confirm its evolutionary relationship within these clades (Figure 2). Consequently, strain ZMU-32-2-1 was definitively identified as *Phomopsis phyllanthicola* ZMU-32-2-1. For strain ZMU-11, the sequence alignment revealed that the closest related strains were *Diaporthe* sp. 4-4 (accession no. LC933365.1) and *Diaporthe* sp. 4-1 (LC933362.1), both sharing 100% identity, followed by *Diaporthe* sp. Yeh 0011 (MW376496.1), *Diaporthe pseudomangiferae* (MG576128.1), and *Diaporthe pseudomangiferae* (MG576127.1), which all shared 98% identity. Consequently, strain ZMU-11 was definitively identified as *Diaporthe* sp ZMU-11. For strain ZMU-49-3, the sequence alignment revealed that the closest related strains were *Diaporthe phaseolorum* (accession no. JQ814361) sharing 95.24% identity, *Diaporthe melonis* var. *brevistylospora* (AB105147) sharing 95.19% identity, *Diaporthe phaseolorum* var. *sojae* (AY050627) sharing 95.14% identity, and *Phomopsis longicolla* (AY745023) sharing 94.31% identity. Consequently, strain ZMU-49-3 was definitively identified as *Diaporthe phaseolorum* ZMU-49-3. The ITS sequences of strains ZMU-32-2-1, ZMU-49-3, and ZMU-11 have been deposited in the GenBank database under accession numbers PZ729687, PZ903488, and PZ903470, respectively.

**FIGURE 2:**
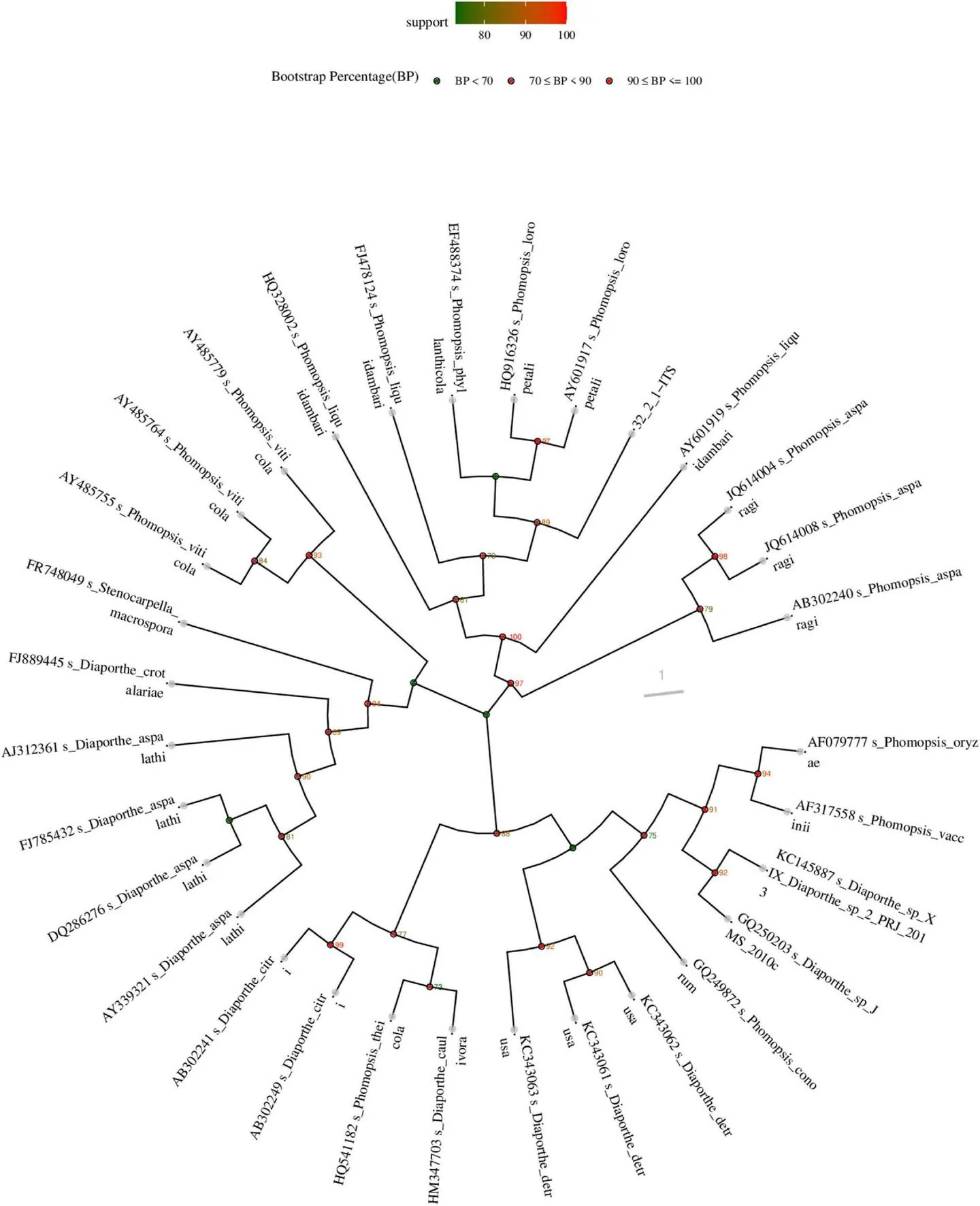
The phylogenetic tree of *P. phyllanthicola* ZMU-32-2-1.

### 3.2 Genome and BGC analysis

Whole-genome sequencing of ZMU-11 and ZMU-32-2-1 revealed assembled genome sizes of 53.23 Mb and 53.04 Mb, with GC contents of 53% and 54%, respectively. For ZMU-11, genome assembly using SPAdes, GapFiller, and PrInSeS-G generated 663 contigs with an N50 of 143,324 bp and a maximum contig length of 530,640 bp. Repetitive sequences accounted for 2.32% of the genome, primarily consisting of unknown repeats (1.15%) and simple sequence repeats (0.95%), followed by LTR elements (0.12%) and low-complexity repeats (0.10%). A total of 16,252 protein-coding genes were predicted, occupying 44.57% of the genome, with an average gene length of 1,460.04 bp. Functional annotation showed that 92.88% of the predicted genes matched homologs in the NCBI NR database, while 45.44%, 62.10%, 58.57%, and 23.07% were assigned to KOG, PFAM, GO, and KEGG, respectively. For ZMU-32-2-1, genome survey analysis indicated an estimated haploid genome size of 49.63 Mb with low heterozygosity (0.2139%), and the final assembly produced 1,690 contigs with an N50 of 58,929 bp and a maximum contig length of 428,238 bp. Repetitive sequences accounted for 1.90% of the genome, mainly comprising simple sequence repeats (0.94%) and unknown repeats (0.85%), with minor contributions from low-complexity repeats (0.09%) and LINE/Jockey elements (0.0083%). A total of 16,155 protein-coding genes were predicted, representing 50.33% of the genome, with an average gene length of 1,652.20 bp. Functional annotation revealed that 97.28% of the predicted genes had matches in the NCBI NR database, whereas 42.81%, 46.79%, 24.52%, and 16.44% were assigned to KOG, PFAM, GO, and KEGG, respectively. In addition, 2.69% of the genes in 32-2-1 were annotated in CAZy, while 0.43%, 1.08%, and 0.27% were assigned to VFDB set A, VFDB set B, and CARD, respectively, indicating the presence of genes potentially related to carbohydrate metabolism, virulence, and drug resistance.

AntiSMASH analysis revealed that ZMU-32-2-1 and ZMU-11 harbor 154 and 123 biosynthetic gene clusters (BGCs), respectively (Figure 3). Both strains exhibit a diverse array of BGC classes, predominantly polyketide synthases (PKS), non-ribosomal peptide synthetases (NRPS and NRPS-like; 31 and 23 clusters in ZMU-32-2-1 and ZMU-11, respectively), terpenes (33 and 10 clusters, respectively), indoles, RiPPs, and hybrid BGCs (e.g., T1PKS-NRPS) (Supplementary Tables 1, 2). Several BGCs in both strains showed high homology to known pathways. In ZMU-32-2-1, cluster 114 (NRPS-T1PKS) and cluster 92 (T1PKS-fungal-RiPP-like) share 96% and 91% similarities with the chaetolivacine and alternapyrone pathways, suggesting the potential production of polyketide antibiotics and azaphilone derivatives, respectively. Additionally, cluster 12 (75% similarity to solanapyrone D) implies phytotoxic potential, while cluster 64 (84% similarity to scytalone/T3HN) suggests a role in melanin biosynthesis. Similarly, ZMU-11 contains clusters with high homology to the fusaristatin A (cluster 106, 90%) and sespendole (cluster 112, 78%) pathways, indicating the capacity to produce antifungal and antimicrobial agents. Other notable BGCs in ZMU-11 include cluster 33 (66% similarity to mycophenolic acid) and cluster 30 (79% similarity to clavaric acid), implying immunosuppressive and antitumor potentials, respectively. Furthermore, both strains possess several toxin-related BGCs, including those homologous to cercosporin (clusters 55 and 88), betaenone A/B/C (clusters 6 and 41), PR-toxin (cluster 45 in ZMU-32-2-1), and zearalenone (cluster 98 in ZMU-11). Notably, approximately 78% of the BGCs in ZMU-32-2-1 and 81% in ZMU-11 showed no significant homology to any known clusters, strongly suggesting the presence of novel biosynthetic pathways. These findings demonstrate that both strains exhibit extensive secondary metabolic diversity, highlighting their substantial potential as promising sources of novel bioactive compounds for pharmaceutical applications.

**FIGURE 3:**
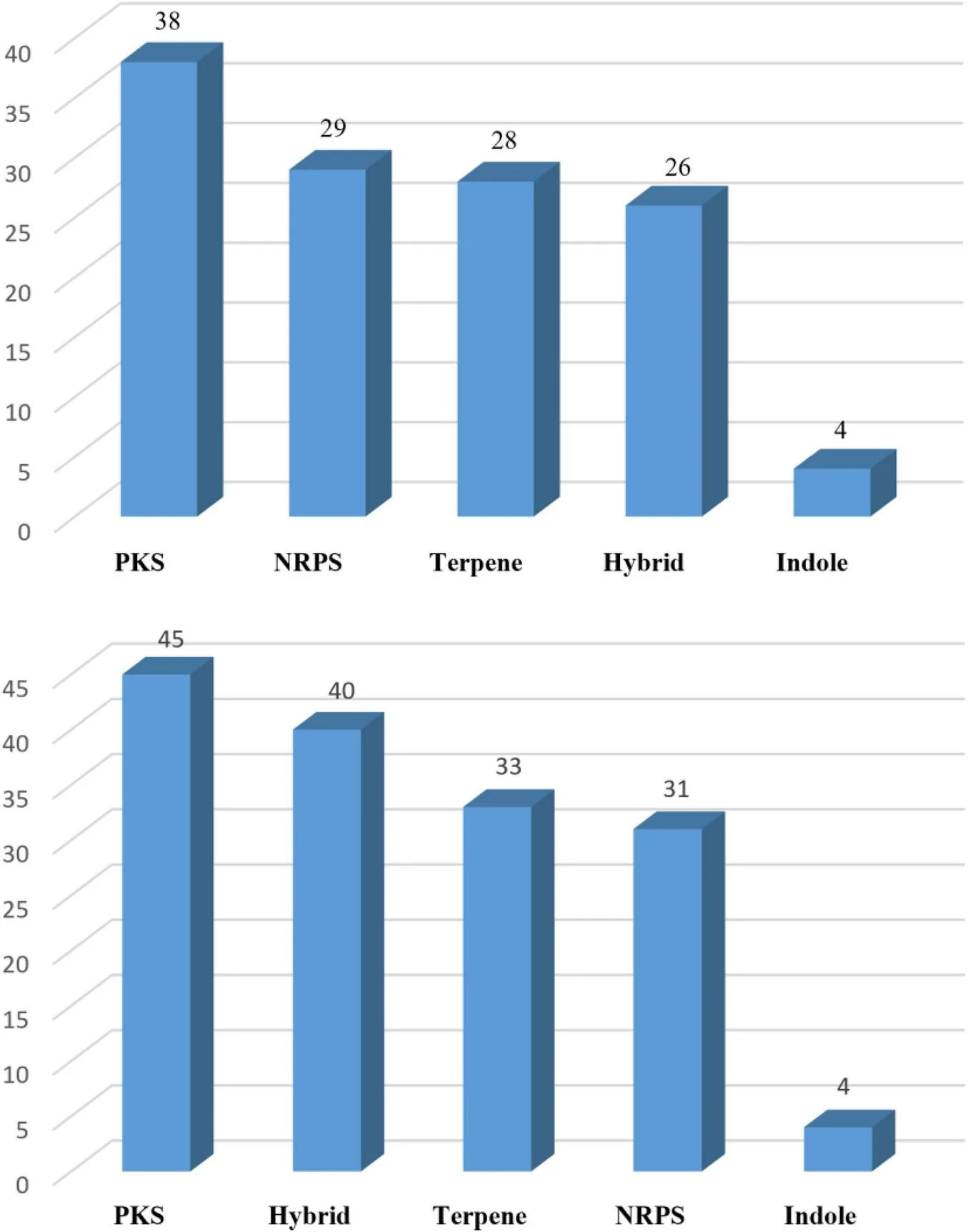
Distribution of different types of BGCs in ZMU-11 (up) and ZMU-32-2-1 (down).

### 3.3 Fungal co-cultivation and HPLC analysis

After isolation and purification, all four fungal strains (ZMU-32-2-1, ZMU-49-3, ZMU-48-1, and ZMU-11) exhibited robust growth (Figure 4A). To evaluate interspecific interactions targeting the selected strain ZMU-11, three tripartite combinations were designed, each containing ZMU-11 together with two of the other three strains: (i) ZMU-32-2-1, ZMU-49-3, and ZMU-11; (ii) ZMU-32-2-1, ZMU-48-1, and ZMU-11; and (iii) ZMU-49-3, ZMU-48-1, and ZMU-11. Compared with the mono-cultures, the co-culture of ZMU-32-2-1, ZMU-48-1, and ZMU-11 caused marked morphological alterations in ZMU-11, including a pronounced reduction in colony diameter, strongly suppressed aerial mycelium development, and a clear inhibition zone between the colonies of ZMU-11 and ZMU-48-1 (Figure 4A). As ZMU-11 showed the most pronounced growth inhibition in this combination, it was provisionally selected as the optimal co-culture system for subsequent investigation.

**FIGURE 4:**
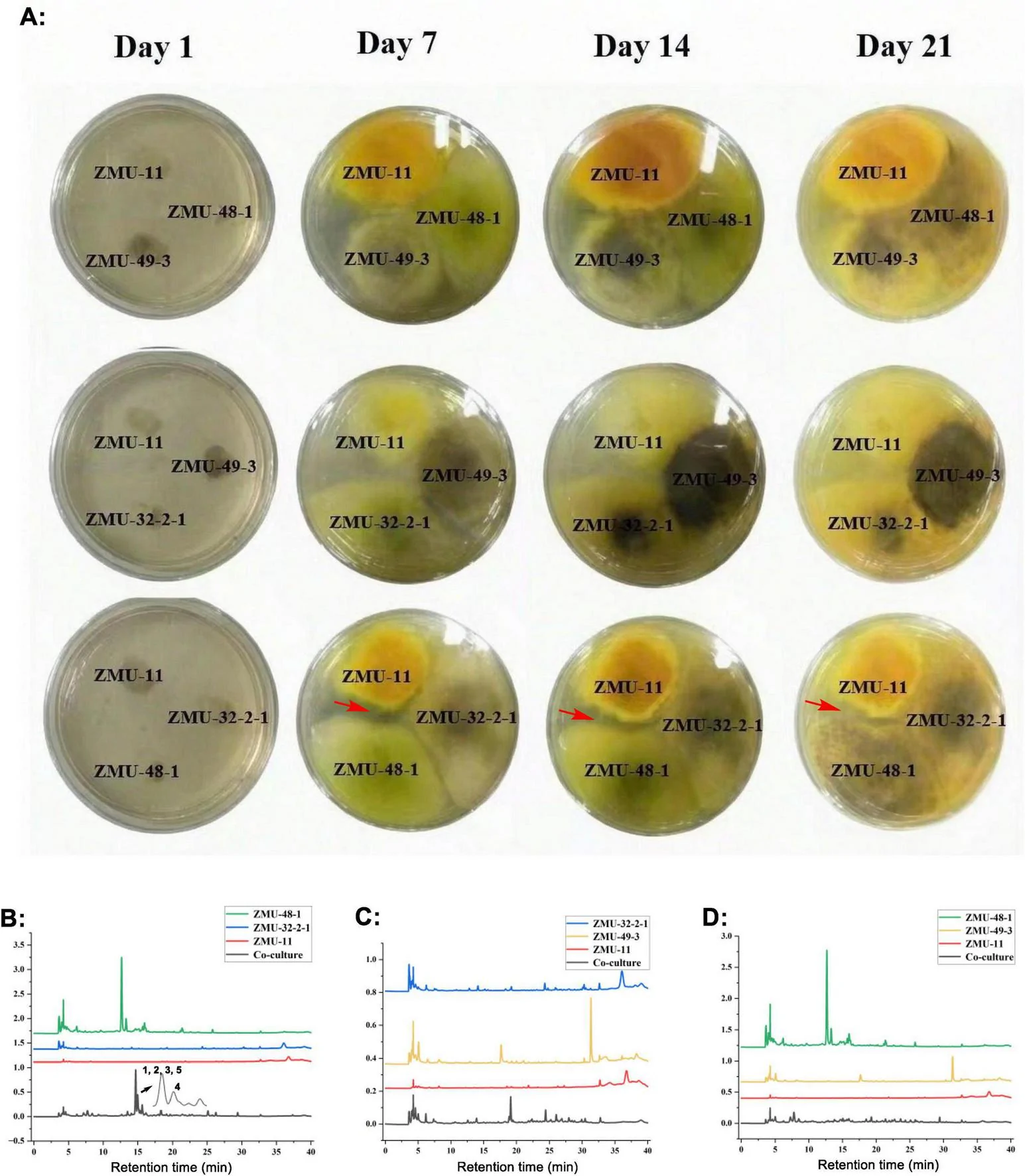
Co-culture combination analysis on PDA plates and HPLC analysis of different co-culture combinations at 254 nm. **(A)** Co-culture combination analysis on PDA plates; **(B)** co culture of ZMU-32-2-1, ZMU-48-1, and ZMU-11; **(C)** co culture of ZMU-32-2-1, ZMU-49-3, and ZMU-11; **(D)** co culture of ZMU-48-1, ZMU-49-3, and ZMU-11. *x*-axis, retention time (min); The chromatograms in panels **(B–D)** are presented in a vertically offset arrangement for comparison; *y*-axis, relative UV absorbance at 254 nm; *x*-axis, retention time (min).

The metabolic profiles of the different co-culture combinations were analyzed by HPLC (Figures 4B–D). Compared with the mono-cultures and the other co-culture combinations, the tripartite co-culture of ZMU-32-2-1, ZMU-48-1, and ZMU-11 exhibited markedly higher metabolite diversity, and several new peaks that were absent from the corresponding mono-cultures appeared at approximately 15 min. In addition, differential signals at approximately 7.5 min were consistently detected in the co-cultures containing both ZMU-11 and ZMU-48-1 (Figures 4B, D), whereas they were absent from the co-culture shown in Figure 4C, further indicating that the coexistence of ZMU-11 and ZMU-48-1 is associated with the differential production or accumulation of specific metabolites. However, owing to their relatively low abundance, these minor signals could not be isolated in sufficient amounts for structural characterization even after scale-up fermentation and were therefore not pursued further. Conversely, certain metabolites detected in the mono-cultures were no longer observed under co-culture conditions, such as the early-eluting peaks (approximately 13 min) of ZMU-48-1 and the signals between 30 and 32.5 min of ZMU-49-3. Taken together, the induction of new metabolites and the suppression of mono-culture-derived signals illustrate the bidirectional regulation of fungal secondary metabolism by co-cultivation: competitive pressure may redirect biosynthetic resources toward defense-related pathways, whereas partner strains may simultaneously biotransform or consume the metabolites produced in mono-culture.

### 3.4 Structural elucidation of the new compounds

Compound 1 was isolated as a white powder, whose molecular formula was determined to be C_14_H_13_NOS on the basis of HR-ESI-MS at m/z 244.0778 [M+H]^+^ (calcd for C_14_H_14_NOS^+^, 244.0791), indicating nine degrees of unsaturation (Supplementary Figure 2). The IR spectrum of compound 1 displayed absorption bands at 2926 and 2851 cm^–1^, attributable to aliphatic C-H stretching vibrations. A strong absorption at 1656 cm^–1^ indicated the presence of a conjugated carbonyl group. The bands at 1581 and 1534 cm^–1^ were consistent with the skeletal vibrations of aromatic and heteroaromatic rings, including C = C and C = N stretching modes (Supplementary Figure 3). The ^1^H NMR spectrum of 1 revealed two sets of singlets (δ_*H*_ 2.26 s; 2.56 s) with an integral area ratio of 3:3 in the high-field region, which suggested the presence of two methyl groups connected to quaternary carbons or heteroatoms. Five sets of aromatic hydrogen signals were also presented, three of which (δ_*H*_ 7.74 d, 2H; 7.70 t, 1H; 7.56 dd, 2H) could be assigned to a monosubstituted benzene ring on the basis of their unique signal features (Yin et al., 2021). The ^13^C NMR spectrum revealed twelve signals, in combination with the HSQC spectrum, which indicated the presence of the above pronounced units, namely, a monosubstituted benzene ring (δ_*C*_ 137.3 s, 133.7 d, 129.7 d, 128.9 d) and two methyl groups (δ_*C*_ 19.3 q, 12.7 q), in addition to a ketone carbonyl group (δ_*C*_ 195.5 s) and a trisubstituted pyridine ring (δ_*C*_ 162.2 s, 149.1 d, 146.6 s, 130.0 s, 122.6 d). In addition, a methyl group was located at C-4 on the basis of the HMBC correlations from H-8 (δ_*H*_ 2.26) to C-3, C-4 and C-5 (Figure 5). A methylthio group was deduced to be connected to C-6 according to the HMBC correction from H-9 (δ_*H*_ 2.56 s, 3H) to C-6 (δ_*C*_ 162.2 s) along with the molecular formula (Ye et al., 2014). Finally, these two rings were connected through the ketone carbonyl bond on the basis of the HMBC correlations from H-2’/H-6’ (δ_*H*_ 7.74 d, 2H) and H-2 (δ_*H*_ 8.34 s, 1H) to the ketone carbonyl carbon at δ_*C*_ 195.5 s. Thus, the structure of 1 was established as diaridine A, and its NMR spectral data are listed in Table 1 and Supplementary Figures 4–8.

**FIGURE 5:**
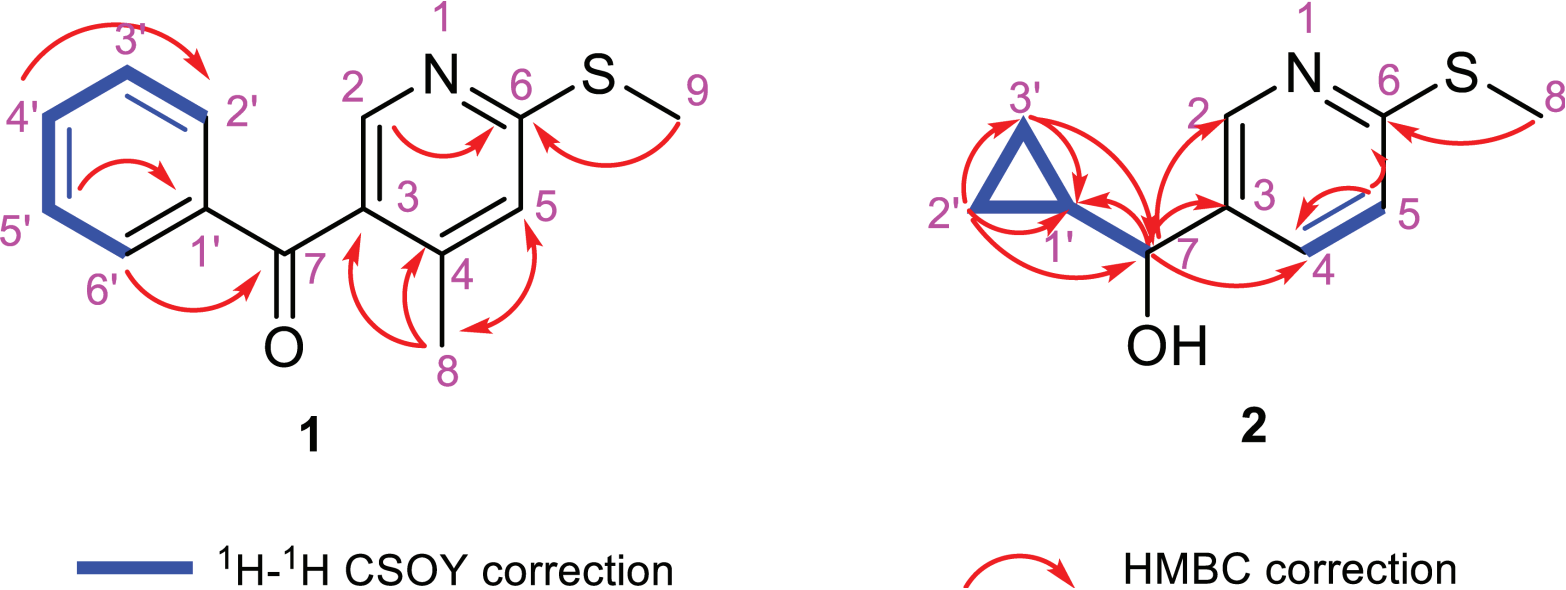
Key HMBC correlations of the new compounds 1–2.

Compound 2 was isolated as a white amorphous powder, whose molecular formula was determined to be C_10_H_13_NOS on the basis of HR-ESI-MS at *m/z* 196.0789 [M+H]^+^ (calcd for C_10_H_14_NOS^+^, 196.0791; Supplementary Figure 9), indicating five degrees of unsaturation. The IR spectrum of compound 2 displayed an absorption band at 3441 cm^–1^, indicating the presence of a hydroxyl group. The absorption bands at 3005 and 2924 cm^–1^ were attributable to C-H stretching vibrations, while the band at 1593 cm^–1^ was consistent with the skeletal vibration of an aromatic/heteroaromatic ring, including C = C and C = N stretching modes (Supplementary Figure 10). The ^1^H NMR spectrum of 2 revealed one singlet methyl signal at δ_*H*_ 2.53 (3H, s), suggesting the presence of a methyl group connected to a heteroatom or quaternary sp^2^ carbon. Three downfield proton signals at δ_*H*_ 8.39 (1H, d, *J* = 2.2 Hz), 7.71 (1H, dd, *J* = 8.3, 2.2 Hz), and 7.25 (1H, d, *J* = 8.3 Hz) were assigned to H-2, H-4, and H-5, respectively, indicating the presence of a trisubstituted pyridine ring. The ^13^C NMR spectrum, in combination with the HSQC data, revealed ten carbon signals, including one methyl carbon (δ_*C*_ 12.1 q), one oxygenated methine carbon (δ_*C*_ 74.9 d), one methine carbon (δ_*C*_ 18.0 d), and two methylene carbons (δ_*C*_ 2.8 t and 1.4 t), in addition to five sp^2^ carbons assignable to a trisubstituted pyridine ring (δ_*C*_ 146.7 d, 136.0 s, 134.5 d, 120.2 d, and 159.1 s). A methylthio group was deduced to be connected to C-6 according to the HMBC correlation from H-8 (δ_*H*_ 2.53, s, 3H) to C-6 (δ_*C*_ 159.1), together with the molecular formula. In addition, the ^1^H-^1^H COSY correlations from H-7 (δ_*H*_ 3.95, d, *J* = 8.3 Hz) to H-1’ (δ_*H*_ 1.13, m), and from H-1’ to H-2’ and H-3’, established the presence of a CH(OH)-cyclopropyl unit. Furthermore, the HMBC correlations from H-7 to C-2, C-3, and C-4, together with the ^1^H-^1^H COSY correlation between H-4 and H-5, supported the assignment of this side chain at C-3 of the pyridine ring (Figure 5). However, the absolute configuration of 2 remained unresolved because its CD spectrum showed no obvious Cotton effect, and the limited sample amount precluded further stereochemical determination. Thus, the planar structure of 2 was established as diaridine B, and its NMR spectral data are listed in Table 1 and Supplementary Figures 11–16.

On the basis of spectroscopic analyses and comparisons with the literature, the known compounds were identified as 12-epicitreoisocoumarinol (3) (Cui et al., 2016), 5-(1-hydroxyhexyl)-6-methyl-2*H*-pyran-2-one (4) (Zhang et al., 2015), and cyclo(_*D*_-*cis*-Hyp-_*L*_-Phe) (5) (Shigemori et al., 1998).

### 3.5 Antifungal activities

The antifungal activities of compounds 1–5 were assessed using MIC method. At an initial screening concentration of 200 μg/mL in a DMSO/water mixture (0.5%–5% *v/v*), none of the compounds exhibited inhibitory activity against the nine tested phytopathogens: *F. graminearum*, *B. dothidea*, *F. oxysporum* Schltdl., *C. gloeosporioides*, *V. mali* Miyabe and G. Yamada, *C. musae*, *A. alternata*, *C. agenarium*, and *B. sorokiniana* (Table 2).

**TABLE 2:** Antifungal activities of compounds 1–5 against plant pathogenic fungi (MIC, μg/mL).

Compounds	*B. sorokiniana*	*B. dothidea*	*F. grami-nearum*	*F. oxysporum* Schltdl	*C. gloeos-porioides*	*V. mali* Miyabe and G. Yamada	*C. musae*	*A. alternata*	*C. agencrium*
MIC, μ g/mL									
1	>200	>200	>200	>200	>200	>200	>200	>200	>200
2	>200	>200	>200	>200	>200	>200	>200	>200	>200
3	>200	>200	>200	>200	>200	>200	>200	>200	>200
4	>200	>200	>200	>200	>200	>200	>200	>200	>200
5	>200	>200	>200	>200	>200	>200	>200	>200	>200
Carbendazim	100	3.125	50	150	1.0625	12.5	6.25	125	12.5

### 3.6 Discussion

In the present study, a cocultivation strategy was employed to explore the cryptic biosynthetic potential of mangrove-derived fungi belonging to the genus *Diaporthe*. The results demonstrated that interspecific interaction markedly altered colony morphology and induced metabolite features not observed in monoculture, underscoring cocultivation as an effective ecological stimulus for activating silent biosynthetic gene clusters (BGCs) in filamentous fungi (Netzker et al., 2015; Yu et al., 2021; Wang et al., 2022; Xu et al., 2023). Ultimately, five metabolites were isolated from the coculture system, including two unprecedented sulfur-containing pyridine alkaloids, diaridines A (1) and B (2), together with three known secondary metabolites. These findings further highlight the substantial metabolic plasticity of *Diaporthe* species in response to microbial interactions. Co-cultivation can activate silent BGCs through multiple biological mechanisms, including: (i) direct physical contact and interspecies chemical signaling (e.g., small diffusible molecules), which may be perceived by cellular receptors and relayed to transcriptional regulators of secondary metabolism; (ii) competition-induced stress responses, such as oxidative stress and nutrient limitation, which are known to trigger epigenetic remodeling (e.g., histone acetylation) and global reprogramming of secondary metabolism; and (iii) cross-talk among the signaling networks of the interacting strains, which may cooperatively shift the metabolic landscape beyond that achievable by any single strain. Consistent with these mechanisms, an obvious antagonistic interaction was observed in the tri-culture of ZMU-48-1, ZMU-32-2-1, and ZMU-11: a clear inhibition zone emerged between the colonies of ZMU-11 and ZMU-48-1, and the growth of ZMU-11 was retarded compared with the other two co-culture combinations (Figure 4A). This visible antagonism provides direct evidence that the co-cultivated strains were engaged in active interspecies competition, which plausibly accounts for the activation of otherwise silent BGCs. Notably, given that diaridines A (1) and B (2) displayed no antifungal activity, their biosynthesis is more plausibly interpreted as part of the stress-responsive metabolic reprogramming elicited by the competitive environment rather than a direct chemical weapon. Although the induced metabolites have been structurally characterized, the specific BGC responsible for diaridines A (1) and B (2) has not yet been identified, since the biosynthetic logic of sulfur incorporation into the pyridine scaffold remains unclear.

Although novel metabolites were successfully induced, none of the isolated compounds exhibited appreciable antifungal activity against the tested phytopathogens at 200 μg/mL. This lack of detectable activity may be attributable to several factors. First, compounds 1 and 2 are relatively small heterocyclic metabolites lacking extended hydrophobic scaffolds, multiple pharmacophores, or highly reactive functionalities that are often required for potent antifungal effects. Second, most of the isolated metabolites were structurally known compounds, suggesting that the coculture condition triggered only a limited subset of the fungal metabolome. Third, antifungal activity is frequently target-dependent and may not be readily manifested under the assay conditions employed. Therefore, the absence of measurable activity should not be interpreted as a lack of biological relevance, but rather as an indication that additional bioassay platforms may be required to reveal other potential pharmacological properties.

The absolute configuration of compound 2 could not be definitively established. Structurally, compound 2 contains only one potential stereogenic center and possesses a relatively flexible side chain, which provides limited conformational restriction for stereochemical assignment. Although circular dichroism (CD) spectroscopy was performed, no obvious Cotton effect was observed. This suggests weak chiroptical response, likely due to the absence of a strong chromophore–stereocenter coupling and conformational averaging in solution (Yamada et al., 2010; Wolf and Bentley, 2013). Under such circumstances, CD analysis is insufficient for unambiguous stereochemical determination. In addition, the limited amount of purified material precluded further stereochemical validation by X-ray crystallography, Mosher ester derivatization, or quantum-chemical ECD calculations. Accordingly, the stereochemistry of compound 2 remains unresolved at present.

Of note, genome mining and antiSMASH analysis revealed that ZMU-32-2-1, ZMU-11 and ZMU-48-1, harbor 154, 123 and 98 BGCs, respectively, the majority of which showed no significant similarity to known clusters. These data strongly suggest that both strains possess a rich yet largely untapped secondary metabolic capacity (Xu et al., 2023). However, only a small number of metabolites were recovered under the present culture conditions, indicating that most BGCs remained transcriptionally silent or expressed at very low levels. Thus, while cocultivation was effective in activating a subset of responsive pathways, it was insufficient to fully unlock the cryptic metabolic space encoded in these fungal genomes. Future studies should therefore combine cocultivation with complementary activation strategies, such as the OSMAC approach, epigenetic modulation, chemical elicitation, signal molecule induction, transcription factor engineering, and heterologous expression (Netzker et al., 2015; Yu et al., 2021; Wang et al., 2022; Xu et al., 2023). Integration of these approaches with transcriptomics and metabolomics would facilitate a more direct linkage between activated BGCs and induced metabolites, thereby improving the efficiency of natural product discovery. Collectively, this work not only yielded two new sulfur-containing pyridine alkaloids from *Diaporthe*, but also reinforced cocultivation as a valuable approach for uncovering cryptic fungal chemistry and expanding the repertoire of fungal natural products.

## 4 Conclusion

In summary, this study highlights the potential of microbial co-cultivation to activate silent biosynthetic pathways in mangrove-derived fungi. Following the isolation and screening of four fungal strains, a tripartite co-culture system comprising ZMU-32-2-1, ZMU-48-1, and ZMU-11 was successfully established. Whole-genome sequencing revealed that these strains harbor a vast array of BGCs. Subsequent HPLC profiling confirmed that this co-culture strategy enhanced the chemical diversity of the secondary metabolome compared to their respective monocultures. Driven by these metabolic variations, large-scale fermentation and chemical purification led to the discovery of two structurally rare sulfur-containing pyridine alkaloids diaridine A (1) and B (2), while the stereochemistry of compound 2 could not be further resolved because of the limited amount of material available. Although these compounds did not exhibit inhibitory activity against nine tested phytopathogens at an initial concentration of 200 μg/mL, the present study suggests that multi-strain co-cultivation may serve as a useful approach for expanding chemical diversity and enabling the discovery of structurally unique metabolites from marine-derived microorganisms.

## Data Availability

The datasets presented in this study can be found in online repositories. The names of the repository/repositories and accession number(s) can be found in the article/Supplementary material.
