# Supplementary material for: Two new sulfur-containing pyridine alkaloids from the co-culture of Diaporthe fungi
Source: Front Microbiol. 2026 Oct 7;17:1963522. doi: 10.3389/fmicb.2026.1963522 (PMC13683570; doi:10.3389/fmicb.2026.1963522)
Supplement: Supplementary file 1 [file Data_Sheet_1.pdf]

Supplementary Material

Two New Sulfur-Containing Pyridine Alkaloids Induced by Co-culture of Three Mangrove-Derived Diaporthe Fungi

Ji-Xin Li[1, +], Yu-Shan Gong[1, +], Meng-Xue Lu[1, 2], Hong-Jie Shen[3], Hao-Xian Lu[3], Xiu-Juan Zhao[1], Tianpeng Yin[1, 2 *], Siwen Yuan[1*]

***Corresponding author**: yuansiwen@zmuzh.edu.cn (SWY); ytp@zmu.edu.cn (TPY)

[+] These authors have made equal contributions to the paper

List of Supporting Information

content

Table S1: ZMU-11 biosynthetic gene cluster statistics........2

Table S2: ZMU-32-2-1 biosynthetic gene cluster statisticse........12

Figure S1: Photographs of the co-cultures of *Diaporthe* strains in liquid medium........23

Figure S2: HRESIMS spectrum of compound 1........24

Figure S3: IR spectrum of compound 1........24

Figure S4: ^{1}H-NMR (600 MHz, CD_3OD) spectrum of compound 1........25

Figure S5: ^{13}C-NMR and DEPT (150 MHz, CD_3OD) spectrum of compound 1........26

Figure S6: HSQC spectrum of compound 1........27

Figure S7: HMBC spectrum of compound 1........28

Figure S8: ^{1}H-^{1}H COSY spectrum of compound 1........29

Figure S9: HRESIMS spectrum of compound 2........30

Figure S10: IR spectrum of compound 2........30

Figure S11: ^{1}H-NMR (600 MHz, CD_3OD) spectrum of compound 2........31

Figure S12: ^{13}C-NMR (150 MHz, CD_3OD) spectrum of compound 2........32

Figure S13: HSQC spectrum of compound 2........33

Figure S15: HMBC spectrum of compound 2........34

Figure S16: ^{1}H-^{1}H COSY spectrum of compound 2........35

Table S1: ZMU-11 biosynthetic gene cluster statistics

Cluster	Type	From	To	Most similar known cluster	Similarity
1	T3PKS	98,391	159,719	NA	
2	other	273,247	334,161	NA	
3	terpene	41,049	71,693	NA	
4	terpene	132,021	163,206	flavunoidine	60%
5	T1PKS,NRPS	218,730	305,067	NA	
6	terpene	1	25,712	NA	
7	T1PKS	184,320	267,749	cryptosporioptide B/cryptosporioptide A/cryptosporioptide C	68%
8	T1PKS	229,976	297,803	NA	
9	NRPS	109,898	176,803	NA	
10	terpene,T1PKS	257,466	338,847	viridicatumtoxin	45%
11	NRPS-like	71,626	135,436	NA	
12	T1PKS	1,984	69,603	NA	
13	T1PKS	91,491	159,572	NA	
14	terpene	173,306	205,556	NA	

15	fungal-RiPP	94,463	146,928	NA	
16	NRPS	1	54,750	NA	
17	NRPS,T1PKS	67,341	181,181	NA	
18	terpene	257,683	289,947	NA	
19	T3PKS	57,920	119,410	NA	
20	T1PKS	168,182	236,255	nectriapyrone C/nectriapyrone D/nectriapyrone	76%
21	isocyanide	116,359	178,471	NA	
22	NRPS-like,T1PKS	181,083	249,868	NA	
23	T1PKS	219,764	287,794	NA	
24	T1PKS	20,056	88,051	NA	
25	NRPS	114,163	181,060	hexadehydroastechrome/terezine-D/astechrome	66%
26	T1PKS	17,440	116,044	harziphilone/t22azaphilone/isoharziphilone-1/isoharziphilone-2/compound 4/compound 1	55%

27	T1PKS	102,819	224,202	duclauxin	68%
28	T1PKS	108,976	174,739	NA	
29	terpene	177,538	208,467	NA	
30	terpene	1	28,723	clavaric acid	79%
31	T1PKS,terpene	29,321	109,651	NA	
32	NRPS,T1PKS	1	54,285	NA	
33	T1PKS	196,610	252,289	mycophenolic acid	66%
34	NRPS-like	98,457	161,712	NA	
35	terpene	129,756	160,624	NA	
36	terpene	41,990	73,174	NA	
37	T1PKS,terpene	101,160	239,624	alternapyrone	75%
38	NI-siderophore	49,683	93,680	NA	
39	terpene	168,499	199,999	NA	
40	T1PKS	116,737	183,414	scytalone/T3HN	84%
41	T1PKS	1	50,653	betaenone A/betaenone B/betaenone C	63%
42	terpene,NRPS-like	118,049	184,198	NA	

43	terpene	97,394	128,468	NA	
44	T1PKS	1	63,300	wortmanamide A/wortmanamide B	74%
45	NRPS	118,888	188,792	NA	
46	terpene	146,238	177,654	NA	
47	terpene-precursor	40,993	72,120	NA	
48	NRPS	118,342	174,489	metachelin C/metachelin A/metachelin A-CE/metachelin B/dimerumic acid 11-mannoside/dimerumic acid	76%
49	indole,terpene, T1PKS	87,125	159,198	NA	
50	terpene	20,651	54,366	NA	
51	T1PKS	89,536	157,130	NA	
52	NRPS-like	137,182	172,024	NA	
53	T1PKS	55,980	125,579	betaenone A/betaenone B/betaenone C	63%

54	indole	49,884	81,349	NA	
55	terpene	118,398	150,360	NA	
56	NRPS-like	36,589	99,851	NA	
57	T1PKS	98,861	151,983	NA	
58	T1PKS	8,363	111,899	chaetoviridin E/11-epichaetomugilin A	NA
59	NRPS	90,495	148,326	NA	
60	NRPS-like	42,726	105,986	NA	
61	T1PKS	80,192	143,668	NA	
62	NRPS,NRPS-like	1	70,403	NA	
63	indole	1	18,838	NA	
64	T1PKS	1	47,580	(-)-Mellein	81%
65	NRPS,betalactone	1	78,846	NA	
66	terpene	21,510	53,022	NA	
67	T1PKS	78,684	133,438	NA	
68	T1PKS,NRPS	36,900	130,181	NA	

69	T1PKS	1	67,089	NA	
70	NRPS	3,754	79,687	NA	
71	NRPS-like	23,092	86,459	NA	
72	NRPS-like	89,408	125,585	NA	
73	NRPS	66,458	124,614	NA	
74	T1PKS	84,681	124,471	NA	
75	terpene	52,536	86,188	NA	
76	NRPS-like	73,787	114,892	NA	
77	terpene	59,871	92,133	NA	
78	terpene	54,815	87,446	NA	
79	terpene,T1PKS	7,828	94,340	alternariol	50%
80	terpene	40,422	96,409	NA	
81	NRPS-like	1	62,279	NA	
82	NRPS,T1PKS	1	46,181	NA	
83	nitropropanoic _acid	56,382	76,518	NA	
84	T1PKS	9,049	76,710	NA	

85	terpene	32,748	65,576	NA	
86	NRPS	10,911	74,643	NA	
87	T1PKS	6,642	74,593	NA	
88	T1PKS	51,737	88,569	cercosporin	67%
89	terpene	18,894	49,997	NA	
90	fungal-RiPP	45,034	83,376	phomopsin A/phomopsin B/phomopsin E	48%
91	NRPS	11,957	76,252	NA	
92	NRPS-like	1	38,528	NA	
93	terpene	1	20,317	NA	
94	NRPS-like	1	35,664	NA	
95	NRP-metallophore, NRPS	1	54,921	NA	
96	terpene	32,952	64,130	NA	
97	NRPS-like	20,239	74,086	NA	
98	T1PKS	1	45,877	zearalenone	66%
99	NRPS,T1PKS	1	70,658	NA	

100	terpene-precursor,NRPS,T1PKS	4,088	70,440	NA	
101	NRPS-like	1	49,894	NA	
102	T1PKS	1	58,327	NA	
103	indole	14,308	45,791	NA	
104	T1PKS	1	62,838	NA	
105	T1PKS	3,513	60,556	orcinol/orsellinic acid	67%
106	NRPS,T1PKS	1	58,218	fusaristatin A	90%
107	T1PKS	9,165	55,549	NA	
108	T1PKS	1	52,846	NA	
109	T1PKS	6,841	52,599	NA	
110	terpene	32,925	52,043	NA	
111	T1PKS	1	46,258	NA	
112	NRPS	1	45,506	enniatin	78%
113	terpene	1	16,843	NA	
114	T1PKS	1	42,255	NA	

115	NRPS,T1PKS	1	41,521	NA	
116	terpene	1	16,382	NA	
117	indole	1	32,731	NA	
118	NRPS-like	1	37,731	choline	79%
119	terpene	2,132	28,854	NA	
120	terpene	10,863	28,325	NA	
121	terpene	1	25,256	α-acorenol	82%
122	T1PKS	1	24,961	NA	
123	NRPS-like	1	18,645	NA	

Table S2: ZMU-32-2-1 biosynthetic gene cluster statisticse

Cluster	Type	From	To	Most similar known cluster	Similarity
1	T3PKS	1	60,639	NA	
2	terpene-precursor	23,705	54,833	NA	
3	terpene	157,924	189,471	NA	
4	T1PKS	153,472	211,084	NA	
5	T1PKS	141,468	192,544	NA	
6	T1PKS	136,488	180,742	BetaenoneA/B/C	64%
7	T1PKS	91,140	159,085	NA	
8	NRPS-like	1	43,047	NA	
9	T1PKS	107,802	153,127	NA	
10	NRPS-like	89,347	147,531	choline	80%
11	NRPS-like	83,027	143,359	NA	
12	T1PKS	63,346	131,663	solanapyrone D	75%
13	fungal-RiPP-like	1	51,948	NA	

14	T1PKS	1	43,755	NA	
15	terpene	69,347	100,859	koraiol	63%
16	terpene	92,241	116,530	NA	
17	NRPS,T1PKS	55,219	115,786	fusaristatin A	89%
18	T1PKS	576	113,773	NA	
19	T1PKS	1	62,941	nectriapyroneC/nectriapyrone D/nectriapyrone	77%
20	terpene-precursor	82,329	106,901	NA	
21	terpene	23,507	55,084	squalestatin S1	62%
22	fungal-RiPP-like	12,003	103,232	NA	
23	NRPS	1	77,212	NA	
24	NRPS-like	5,568	69,052	NA	
25	T1PKS,NRPS	177	86,479	NA	
26	NRPS	1	44,918	hexadehydroastechrome/terezine-D/astechrome	72%
27	terpene	964	32,227	NA	
28	terpene	50,639	83,120	clavaric acid	70%

29	terpene	59,145	90,250	NA
30	NRPS,T1PKS	1	69,980	NA
31	T1PKS,NRPS<br>-like	1	49,483	NA
32	fungal-RiPP-<br>like	1	72,372	NA
33	fungal-RiPP-<br>like	1	53,560	NA
34	NRPS	23,666	86,017	NA
35	NRPS	1	50,063	NA
36	NRPS	24,804	85,129	NA
37	NRPS-like	1	47,630	NA
38	fungal-RiPP-<br>like	1	67,949	NA
39	NRPS,T1PKS	1	60,431	NA
40	fungal-RiPP-<br>like	1	78,356	NA
41	terpene	60,645	77,949	NA
42	T1PKS	1	67,163	NA

43	terpene	5,390	36,661	α-acorenol	65%
44	NRPS-like	19,200	73,751	NA	
45	terpene,T1PKS	38	72,011	PR-toxin	90%
46	NRPS	1	49,997	NA	
47	T1PKS	26,517	71,402	NA	
48	terpene	1	20,083	NA	
49	other	1	44,563	NA	
50	indole	17,536	49,001	NA	
51	terpene	1	21,095	NA	
52	T1PKS	26,714	66,812	NA	
53	T1PKS	10,285	65,929	NA	
54	terpene	13,206	46,034	NA	
55	T1PKS	5,628	63,633	cercosporin	67%
56	terpene	40,413	61,941	NA	
57	T1PKS	20,759	61,877	NA	
58	terpene	19,016	52,222	NA	

59	nitropropanoic_acid	1	13,150	NA	
60	T1PKS	8,800	54,140	NA	
61	NRPS	1	44,385	NA	
62	NRPS-like	6,083	53,713	NA	
63	T1PKS	1	52,981	NA	
64	T1PKS	1	36,920	scytalone/T3HN	84%
65	indole	5,605	37,088	NA	
66	fungal-RiPP-like	1	51,940	NA	
67	NRPS	10,125	51,740	NA	
68	terpene	34,977	51,516	NA	
69	NRPS	1	46,580	NA	
70	T1PKS	9,631	50,710	epipyrone A	76%
71	terpene	1,192	36,570	NA	
72	terpene	26,232	49,902	NA	
73	T1PKS	1	41,614	dichlorodiaporthin	NA

74	T1PKS	9,983	49,696	(-)-Mellein	81%
75	T1PKS,NRPS	1	49,266	NA	
76	T1PKS	1	48,501	chaetoviridin E/11-epichaetomugilin A	NA
77	terpene	1,573	35,904	NA	
78	NRPS-like	1	47,829	NA	
79	T1PKS	1	47,750	harziphilone/t22azaphilone/isoharziphilone-1/isoharziphilone-2/compound 4/compound 1	74%
80	T1PKS	1,280	47,427	NA	
81	fungal-RiPP-like	1	46,682	NA	
82	T1PKS	1	46,563	NA	
83	terpene	1	20,131	NA	
84	NRPS-like	1	36,121	NA	
85	NRPS-like	134	45,441	NA	
86	terpene	18,483	45,001	NA	
87	terpene	24,530	44,676	HEx-tc1 terpene	39%

88	fungal-RiPP-like,terpene	1	43,559	NA	
89	terpene	1	29,625	NA	
90	terpene	1	18,029	NA	
91	indole	2,411	33,187	NA	
92	T1PKS,fungal-RiPP-like	1	42,400	alternapyrone	91%
93	NI-siderophore	19,847	42,359	NA	
94	T1PKS	1	41,361	zearalenone	66%
95	T1PKS	1	41,301	3'-methoxy-1,2-dehydropenicillide/pestalotiollide B/pestalotiollide C/1,2-dehydropenicillide/3-methoxy-1,2-dehydropenicillide/1,2-epoxy-3,4-didehydropenicillide	78%
96	terpene-precursor	22,392	41,150	NA	
97	isocyanide	1	40,138	NA	

98	NRPS-like	3,261	38,631	NA	
99	fungal-RiPP-like	1	38,617	NA	
100	NRPS	1	38,534	metachelinC/metachelinA/metachelinA-CE/metachelinB/dimerumic acid11-mannoside/dimerumic acid	68%
101	NRPS	1	37,763	NA	
102	fungal-RiPP-like,indole	1	36,677	NA	
103	NRPS,fungal-RiPP-like	1	36,062	NA	
104	terpene	12,050	35,751	NA	
105	terpene	12,960	35,692	NA	
106	T1PKS	1	35,482	harziphilone/t22azaphilone/isoharziphilone-1/isoharziphilone-2/compound 4/compound 1	78%
107	fungal-RiPP-like	1	32,808	NA	

108	terpene	10,740	32,760	NA	
109	NRPS	1	32,703	NA	
110	terpene	1	16,721	NA	
111	T1PKS	1	31,661	NA	
112	terpene	9,382	31,652	NA	
113	terpene	1	27,114	NA	
114	NRPS,T1PKS	1	30,657	ChaetolivacineA/chaetolivacineB/chaetolivacine C	96%
115	fungal-RiPP-like	1	30,548	NA	
116	NRPS-like	1	30,403	NA	
117	fungal-RiPP-like	1	30,132	NA	
118	indole	1	19,378	NA	
119	T1PKS,fungal-RiPP-like	1	29,703	NA	
120	NRPS	1	29,339	NA	
121	indole	3,392	28,888	NA	

122	NRPS-like	1	28,790	NA	
123	T1PKS	1	28,694	duclauxin	68%
124	T3PKS	1	28,262	NA	
125	T1PKS	1	28,191	NA	
126	NRPS-like	1	28,149	NA	
127	T1PKS	1	28,025	NA	
128	terpene-precursor	2,846	27,622	NA	
129	T1PKS	1	25,523	NA	
130	fungal-RiPP-like	1	23,673	NA	
131	betalactone	1	23,456	NA	
132	T1PKS	1	23,410	NA	
133	fungal-RiPP-like	1	22,957	NA	
134	T1PKS,NRPS	1	22,743	NA	
135	T1PKS	1	22,380	NA	
136	NRPS	1	22,262	NA	

137	terpene	1	17,217	NA	
138	T1PKS	1	21,189	NA	
139	T1PKS	1	21,010	NA	
140	NRPS,T1PKS	1	19,941	atpenin B	79%
141	terpene	1	18,170	NA	
142	NRPS-like	1	19,619	patulin	NA
143	fungal-RiPP-like	1	19,562	NA	
144	T1PKS,NRPS	1	19,306	NA	
145	T1PKS	1	19,280	NA	
146	terpene	1	18,702	NA	
147	T1PKS	1	18,603	NA	
148	T1PKS	1	17,910	viridicatumtoxin/previridicatumtoxin/5-hydroxyanthrotainin/8-O-desmethylanthrotainin	78%
149	NRPS	1	17,635	NA	
150	NRPS	1	17,502	enniatin	76%

151	T1PKS	1	16,692	NA
152	T1PKS	1	14,502	NA
153	terpene	1	14,486	NA
154	T1PKS	1	14,151	NA

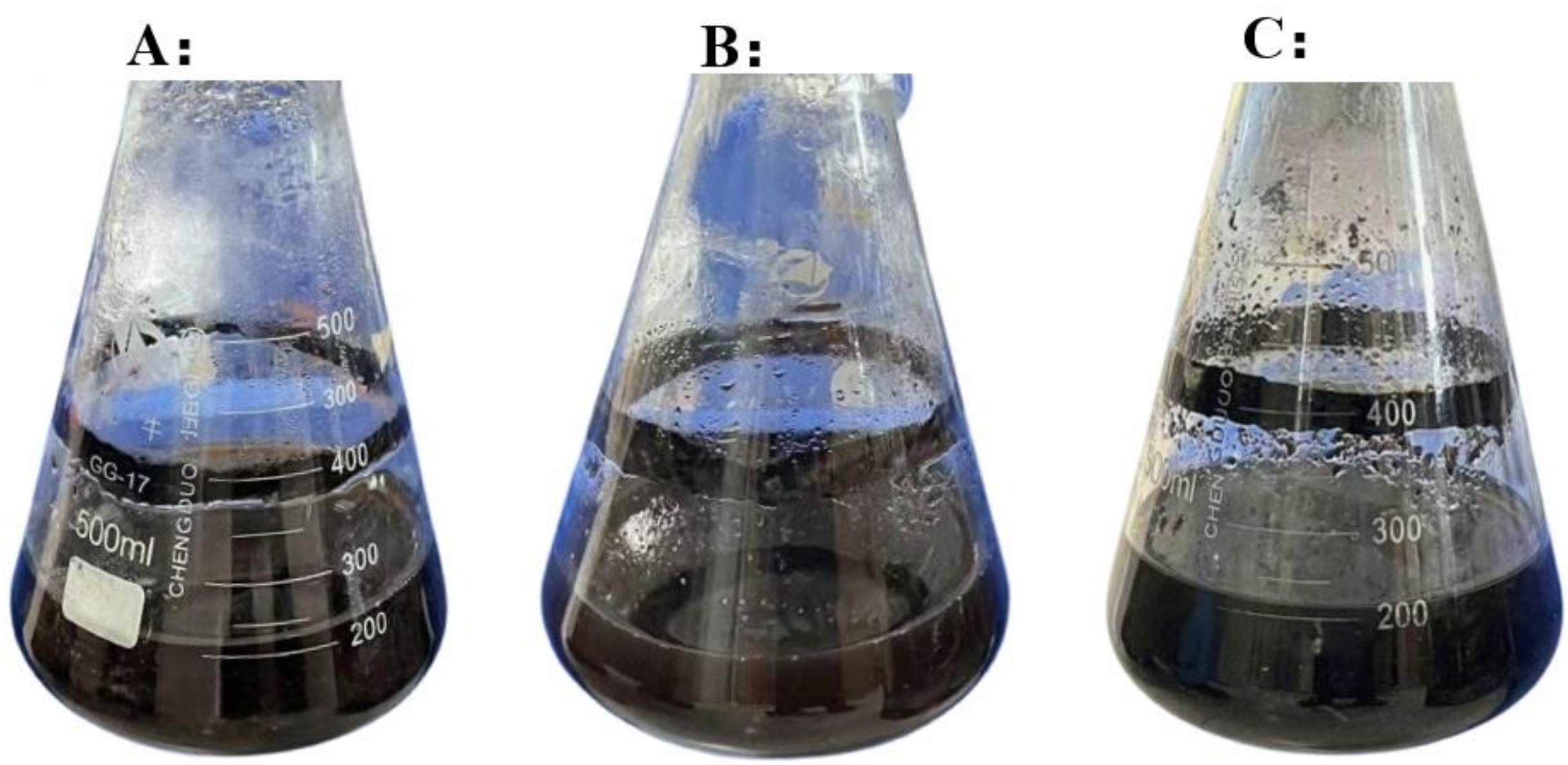

Figure S1: Photographs of the co-cultures of *Diaporthe* strains in liquid medium. The cultures were incubated at 30 °C for 21 days. A: Co-culture of ZMU-32-2-1, ZMU-48-1, and ZMU-11; B: Co-culture of ZMU-32-2-1, ZMU-49-3, and ZMU-11; C: Co-culture of ZMU-48-1, ZMU-49-3, and ZMU-11

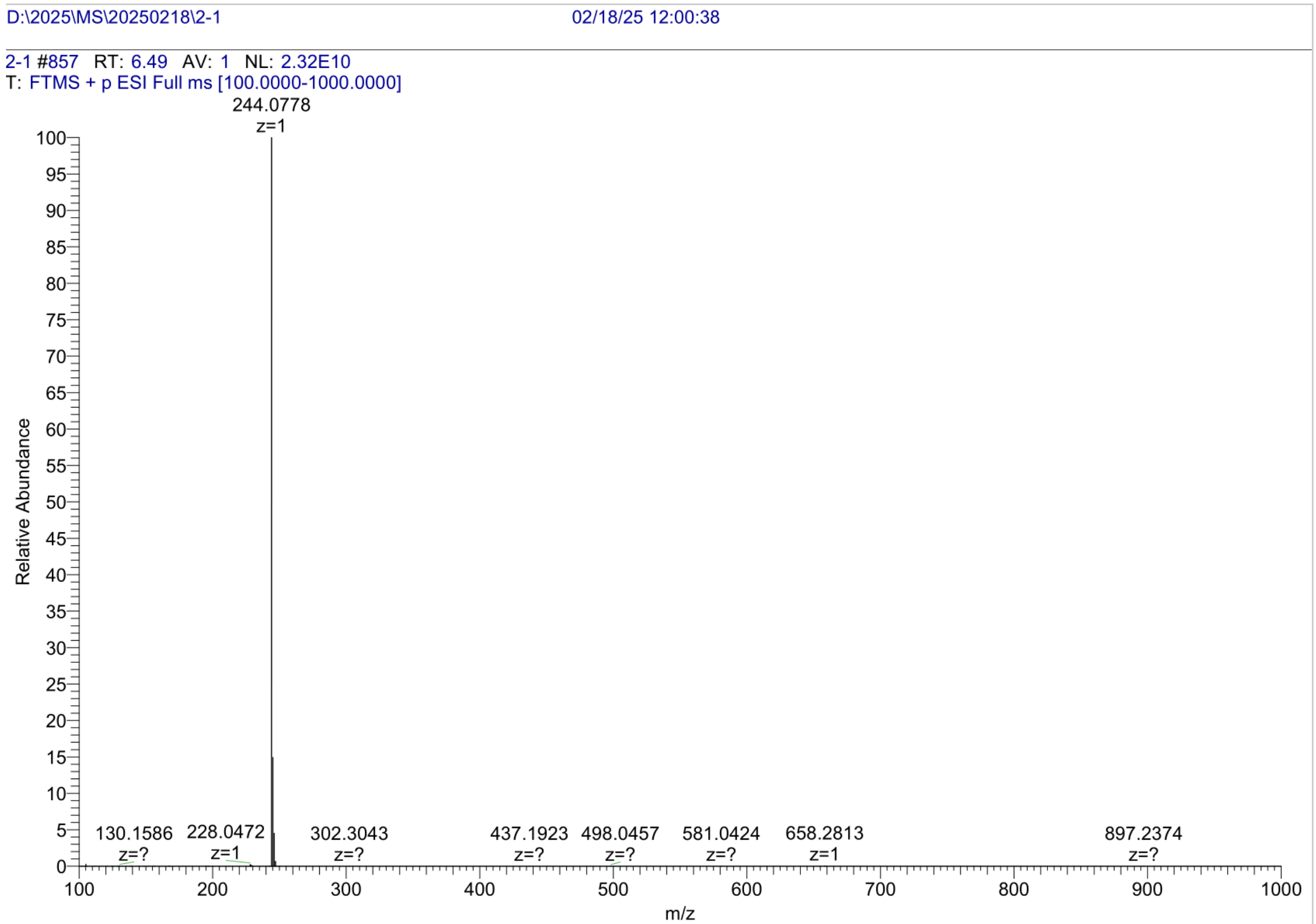

Figure S2: HRESIMS spectrum of compound 1

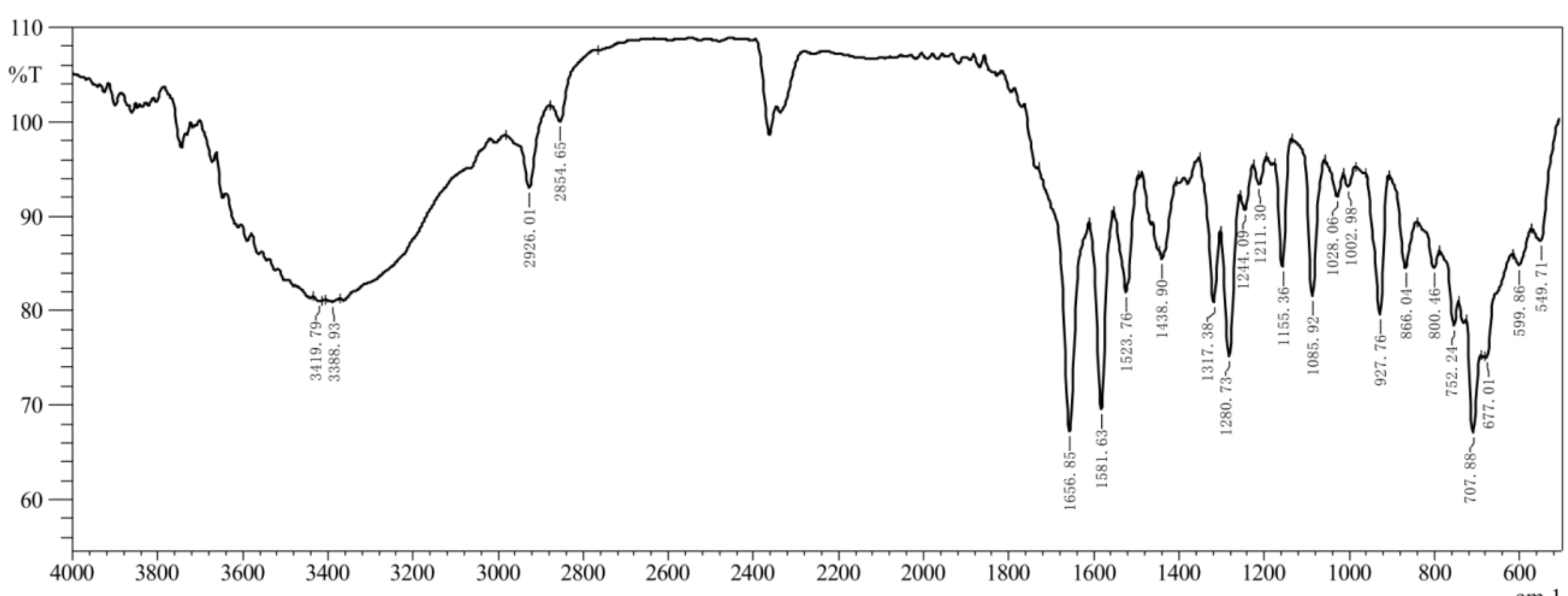

Figure S3: IR spectrum of compound 1

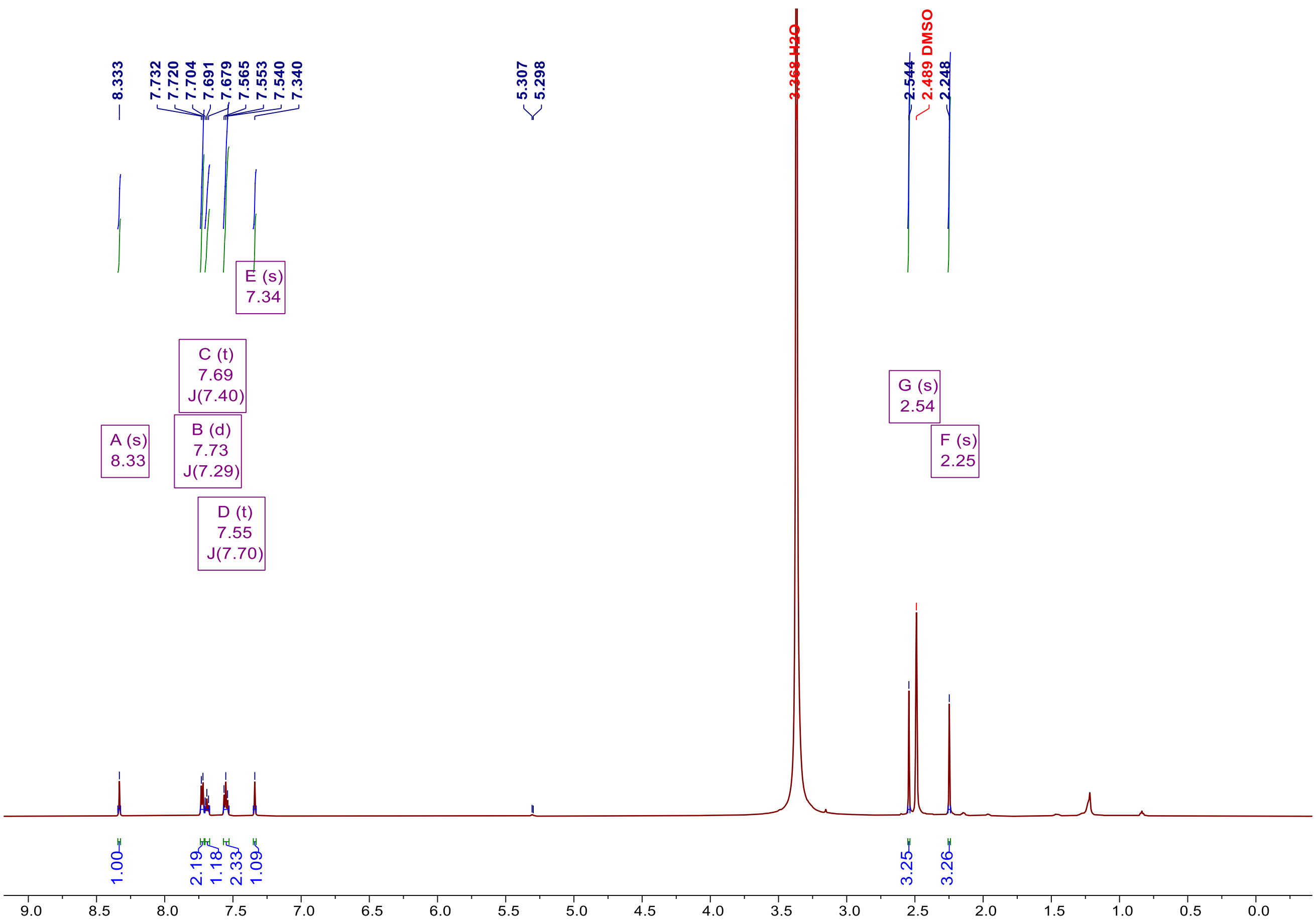

Figure S4: ^{1}H-NMR (600 MHz, CD_3OD) spectrum of compound 1

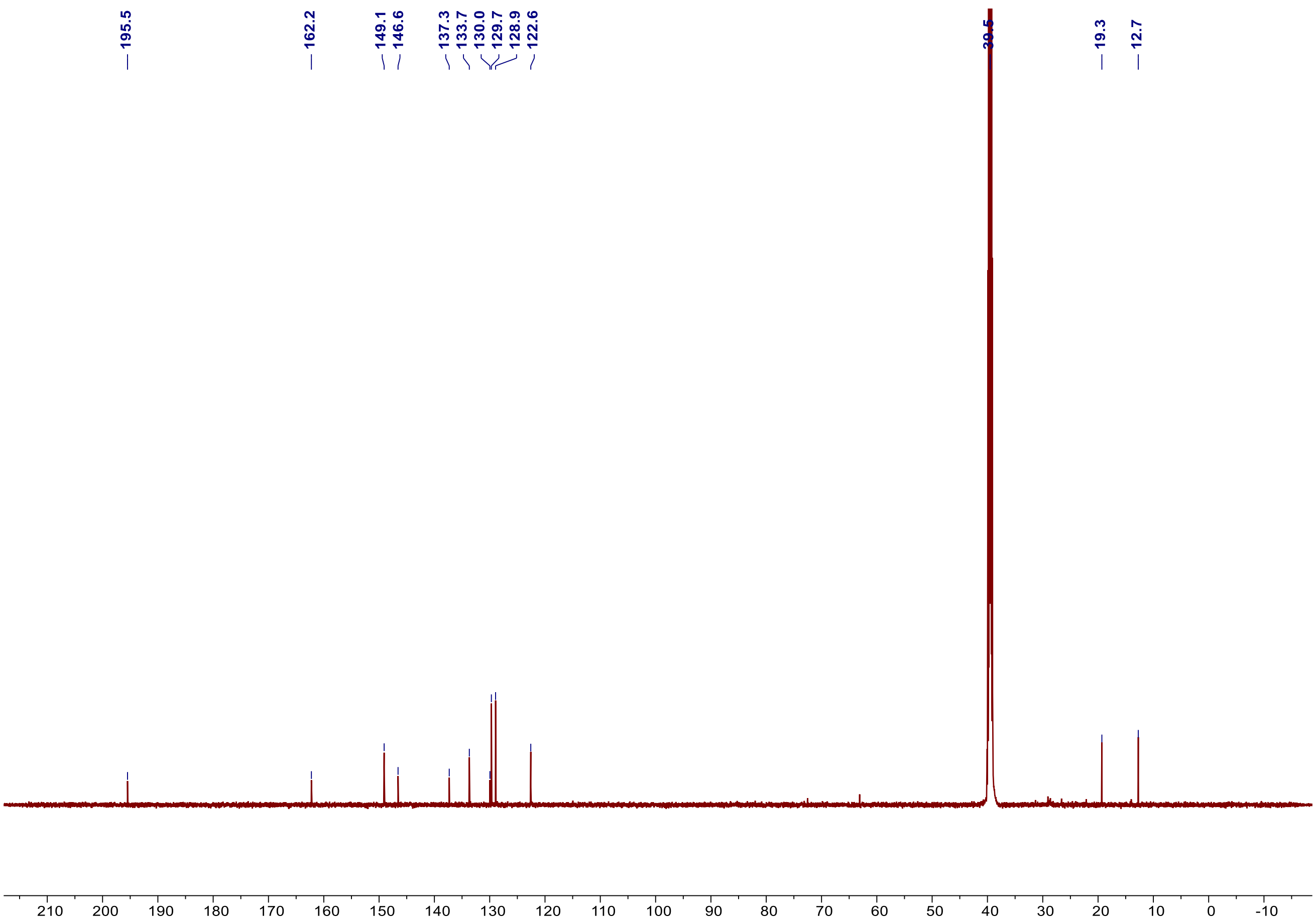

Figure S5: ^{13}C-NMR and DEPT (150 MHz, CD_3OD) spectrum of compound 1

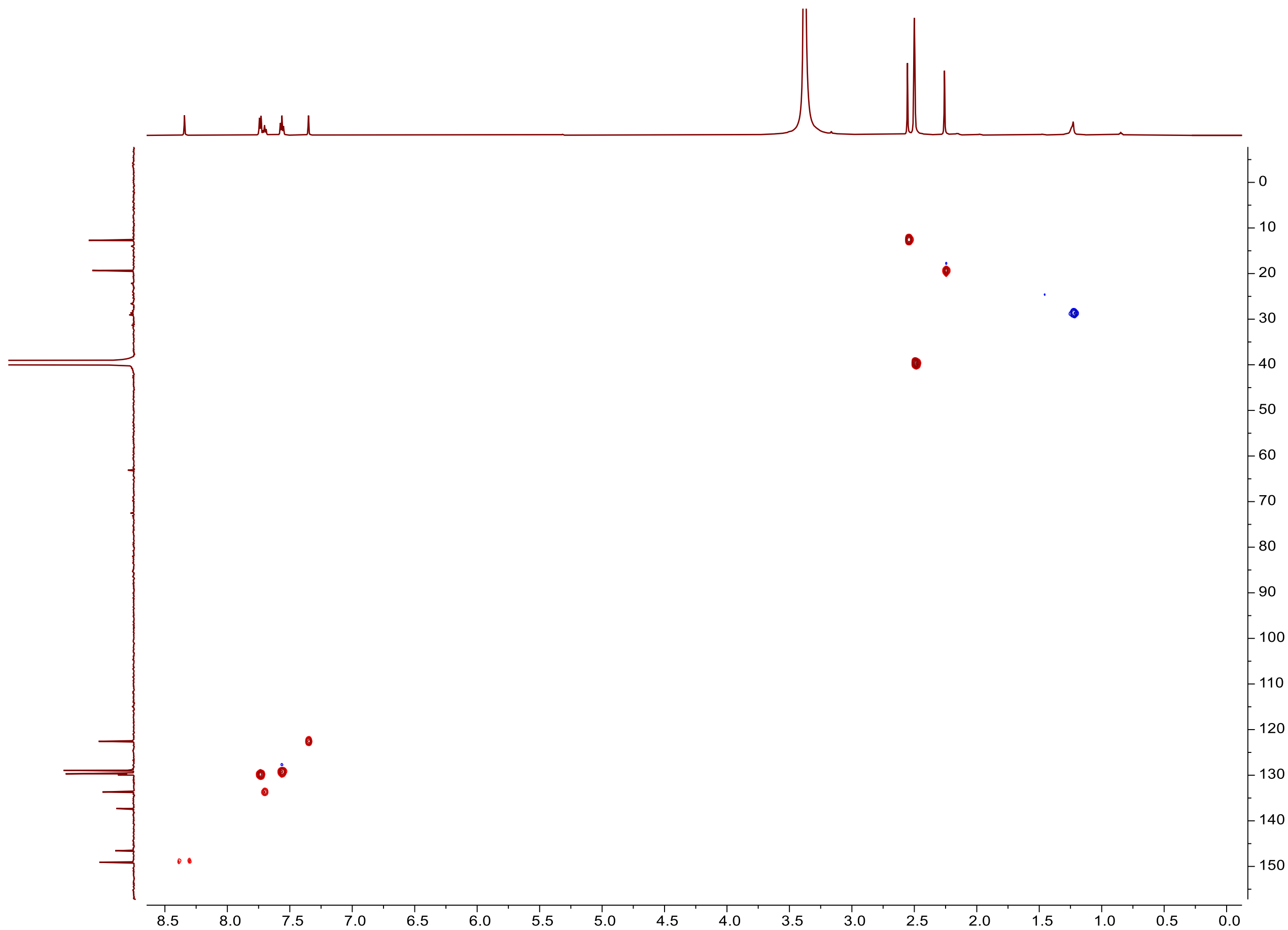

Figure S6: HSQC spectrum of compound 1

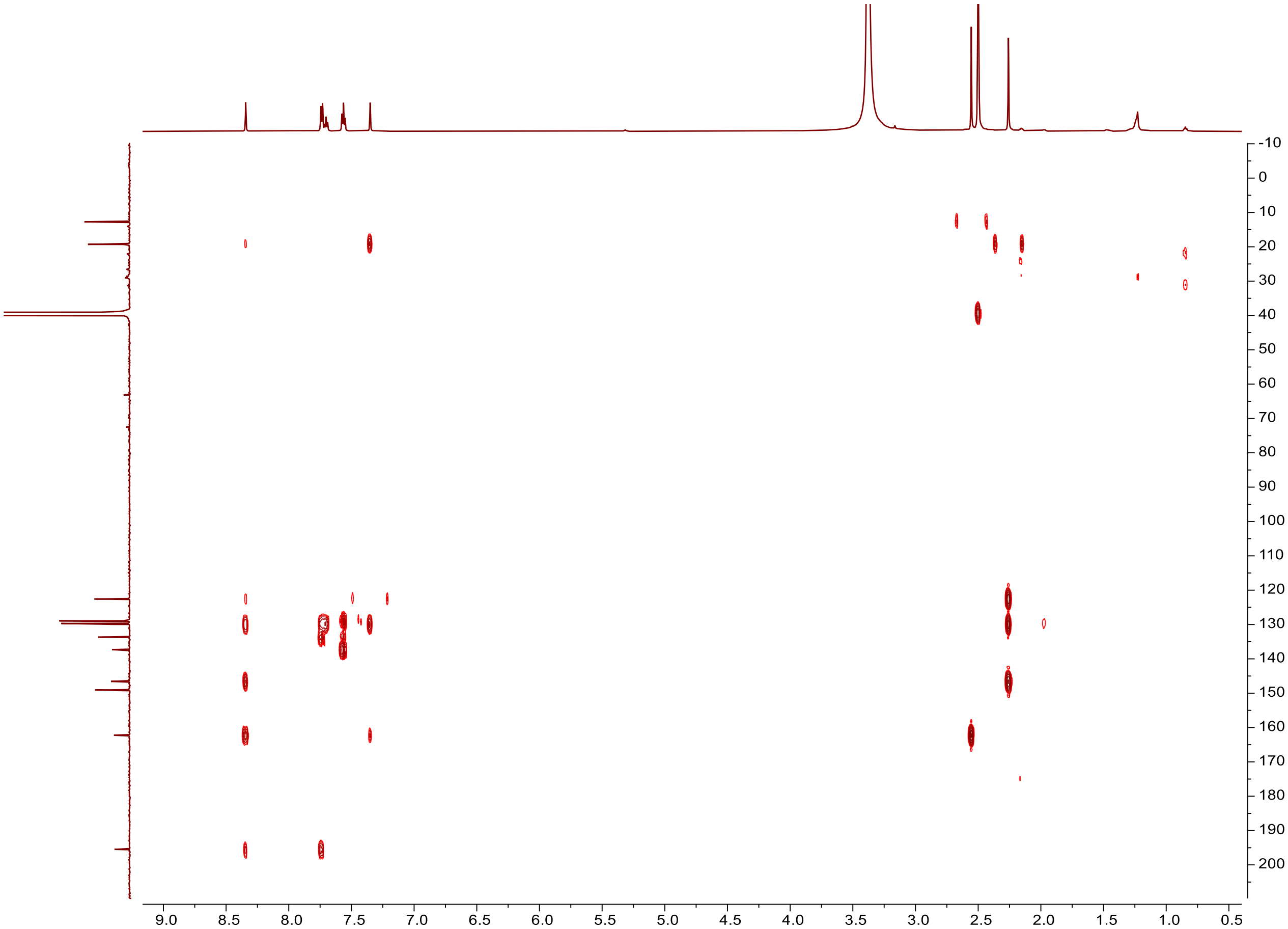

Figure S7: HMBC spectrum of compound 1

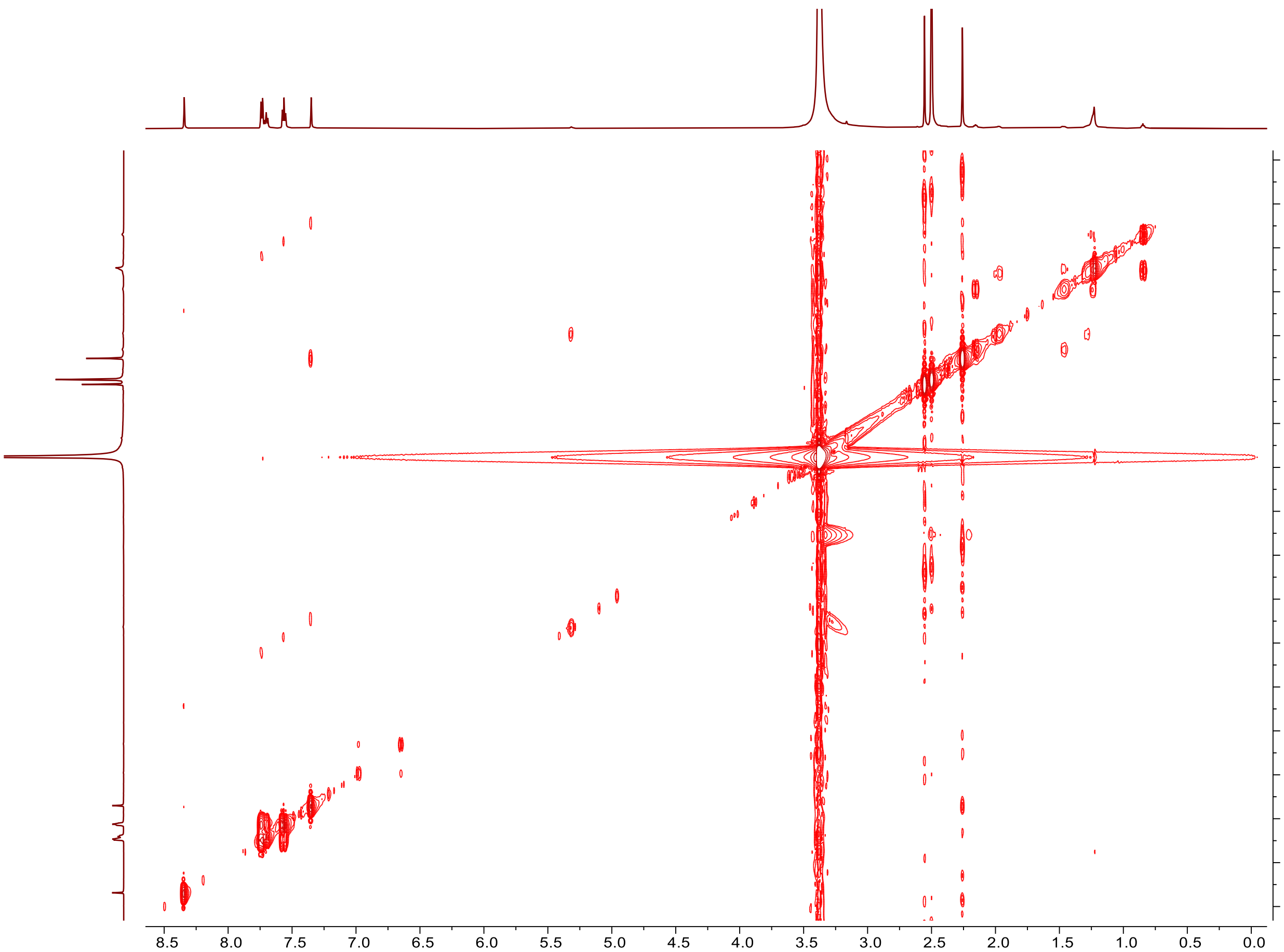

Figure S8: ^{1}H-^{1}H COSY spectrum of compound 1

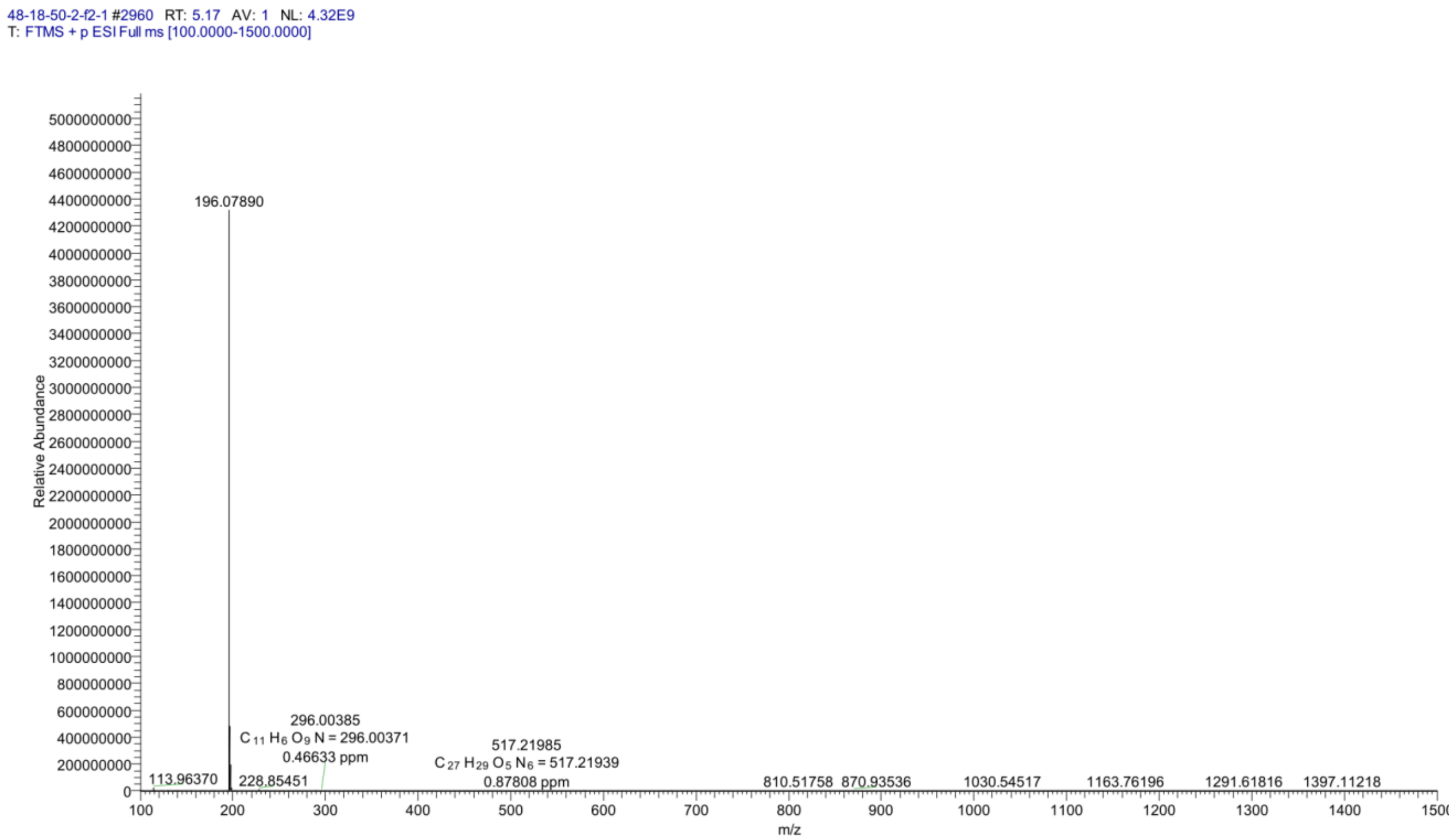

Figure S9: HRESIMS spectrum of compound 2

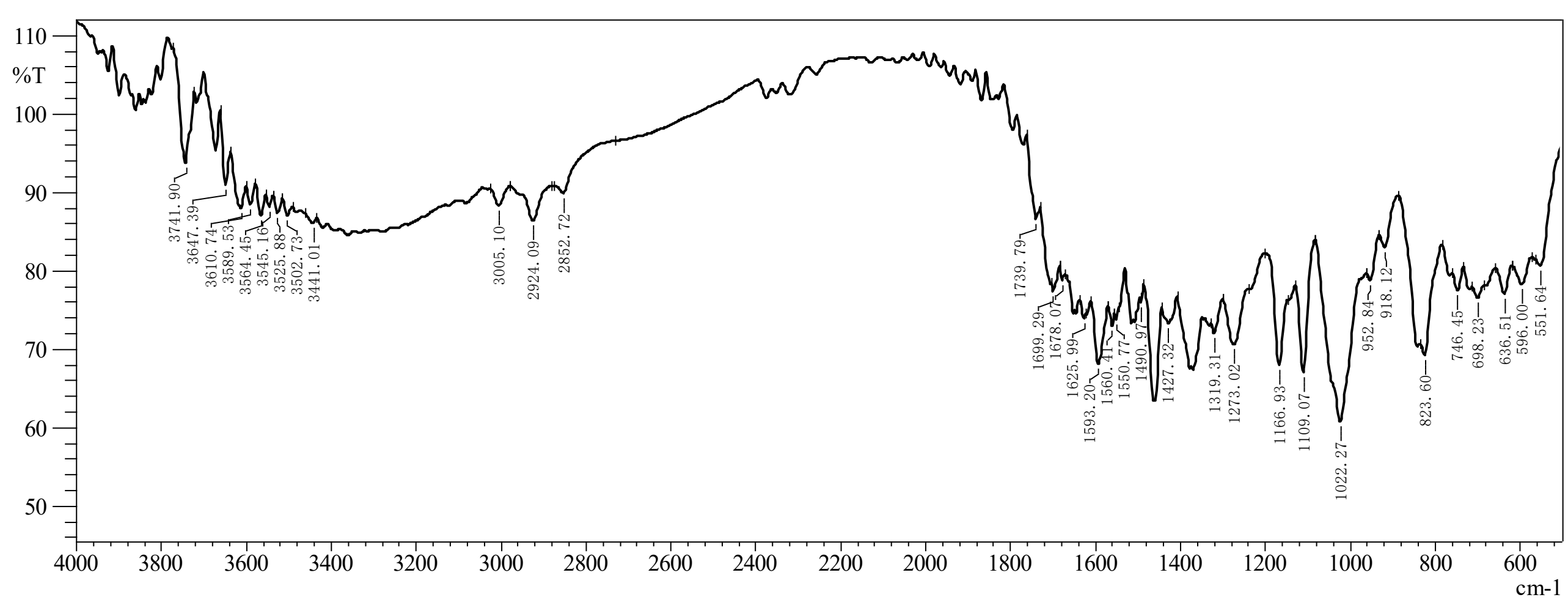

Figure S10: IR spectrum of compound 2

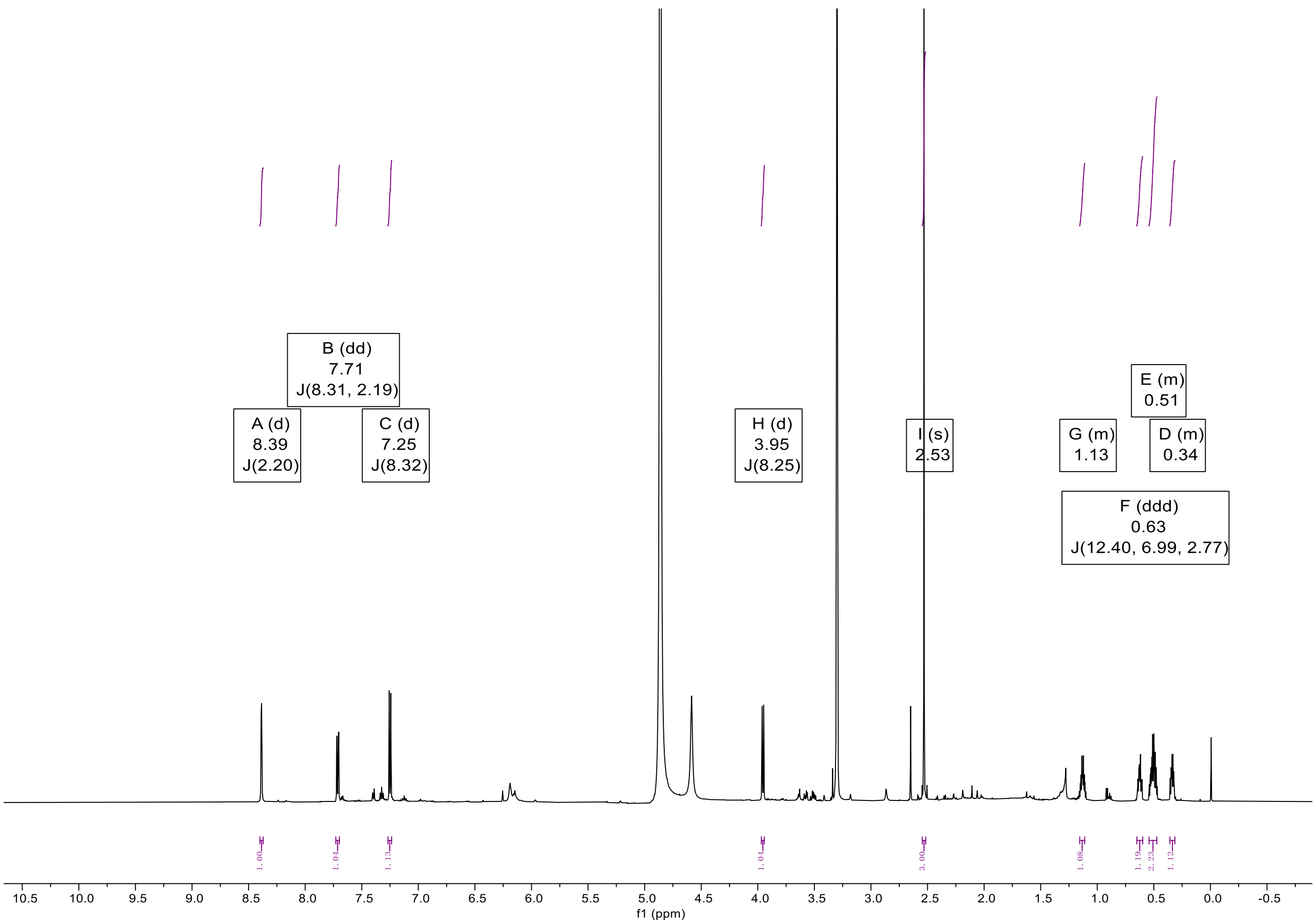

Figure S11: ^{1}H-NMR (600 MHz, CD_3OD) spectrum of compound 2

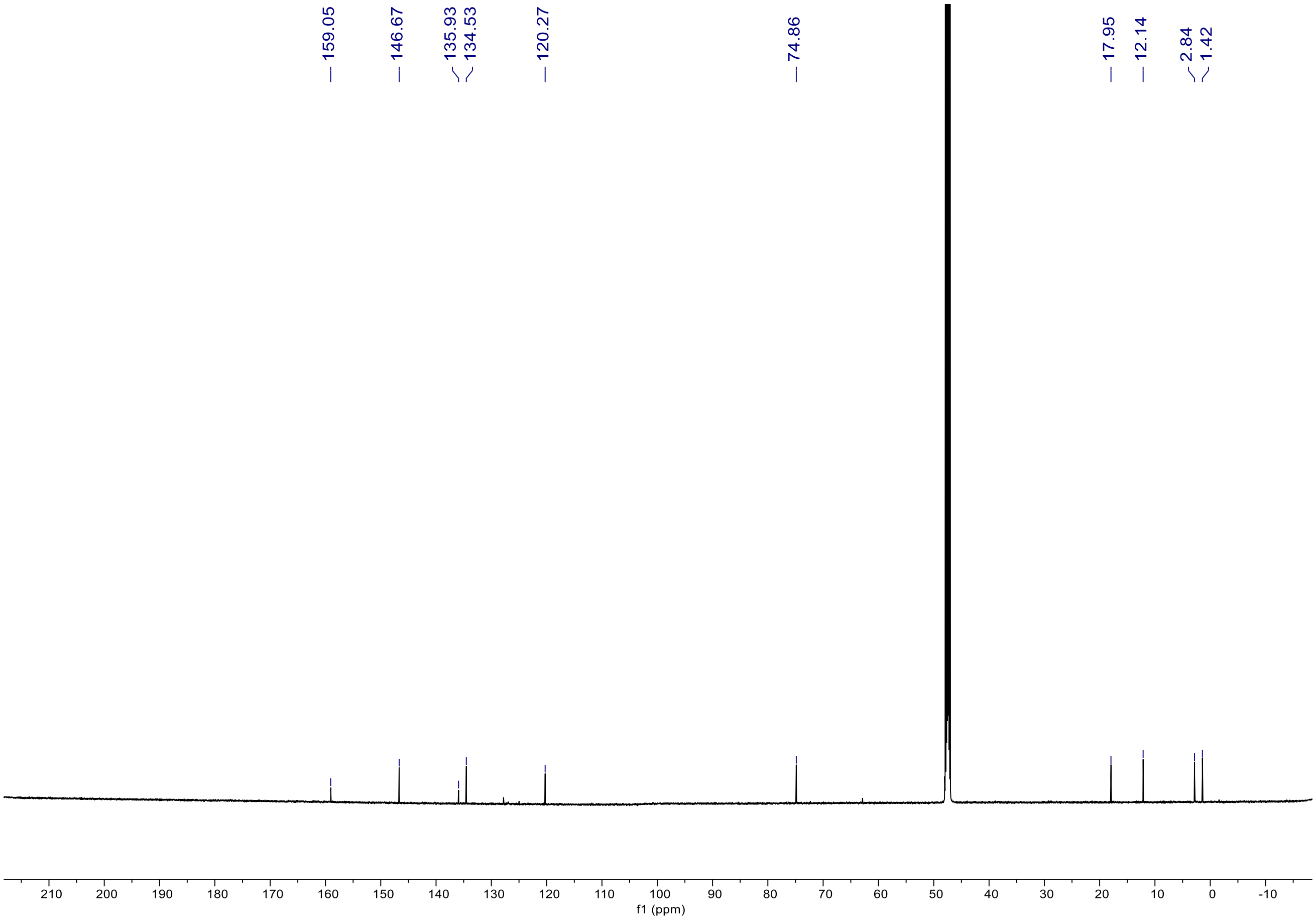

Figure S12: ^{13}C-NMR (150 MHz, CD_3OD) spectrum of compound 2

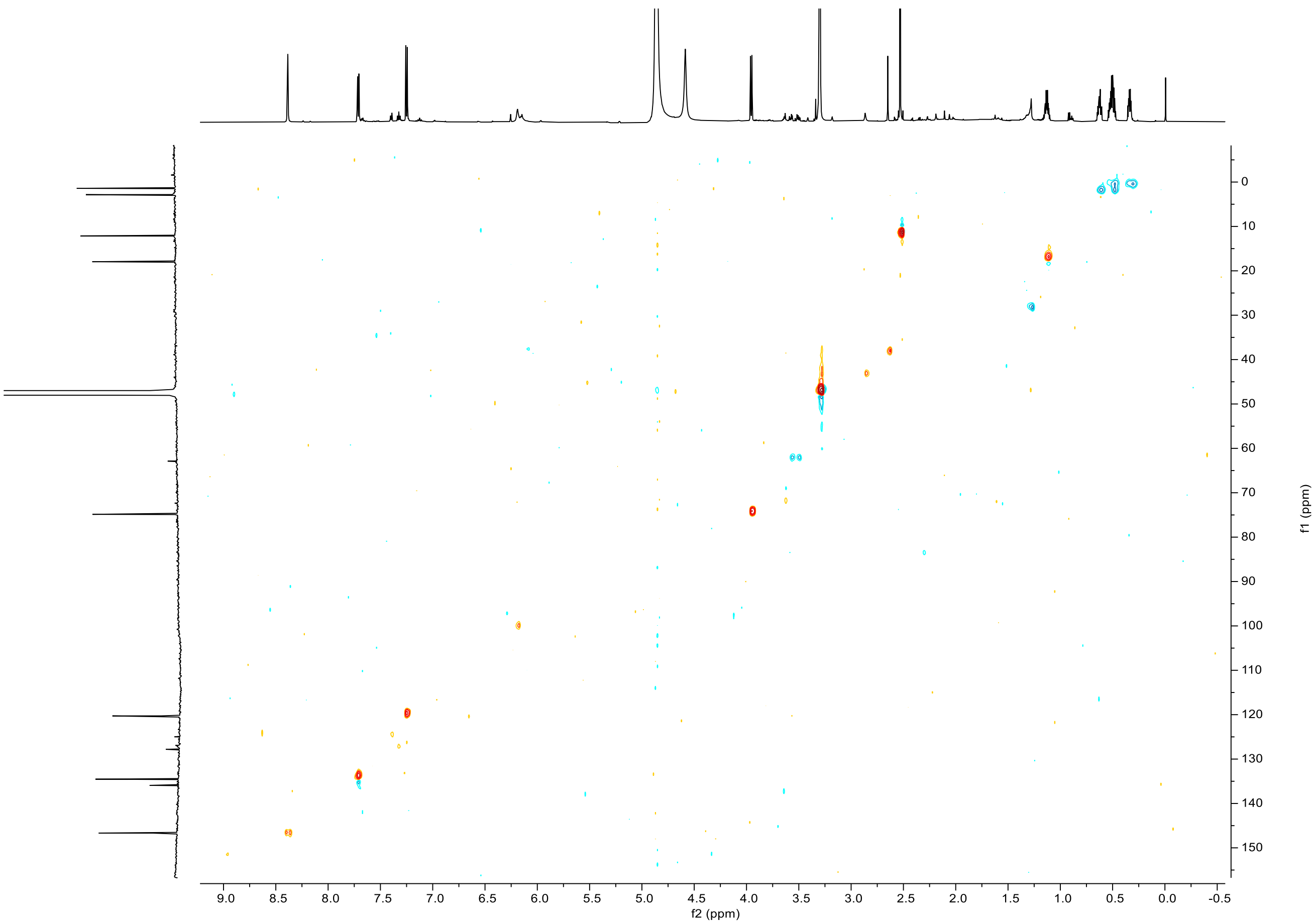

Figure S13: HSQC spectrum of compound 2

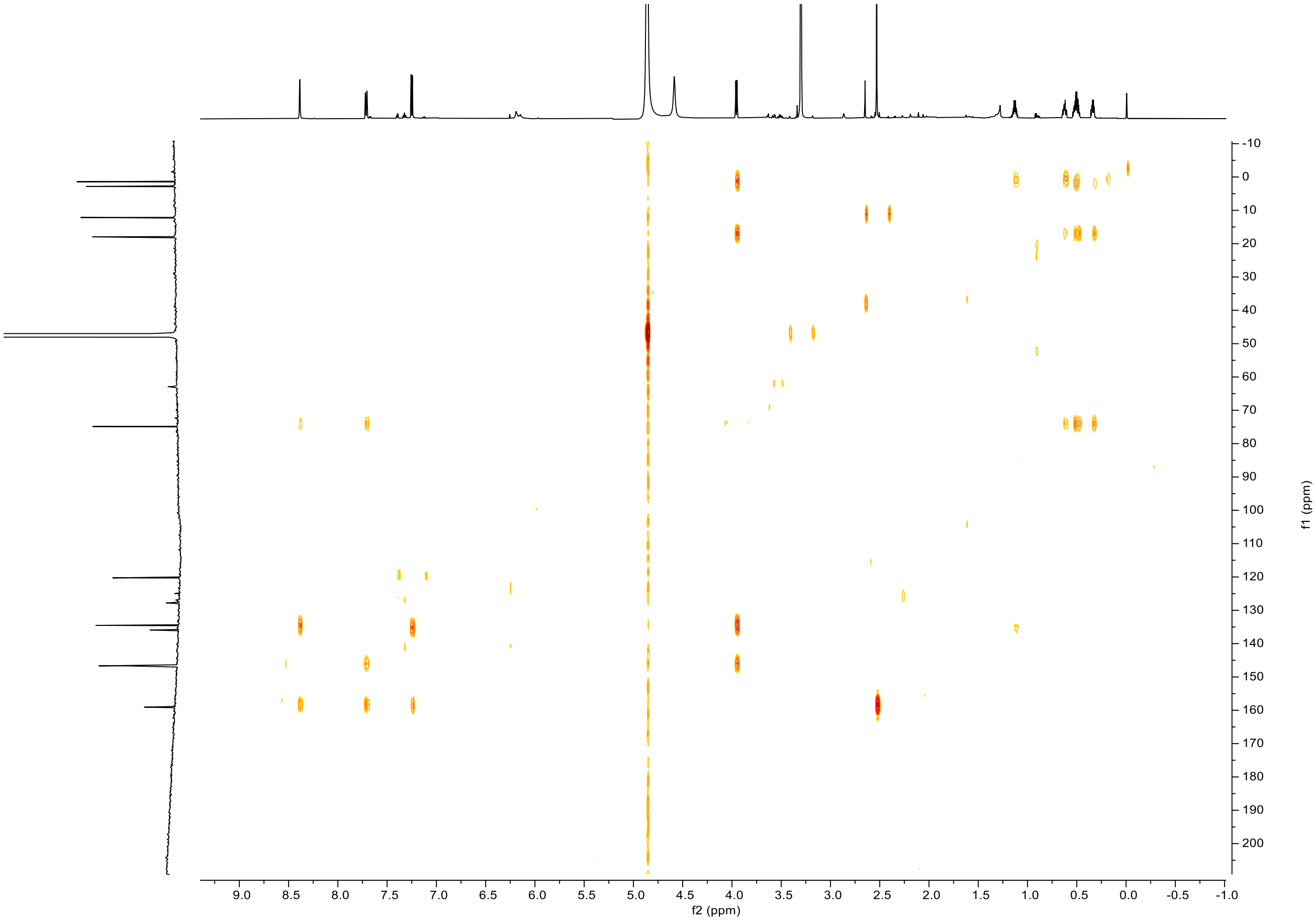

Figure S15: HMBC spectrum of compound 2

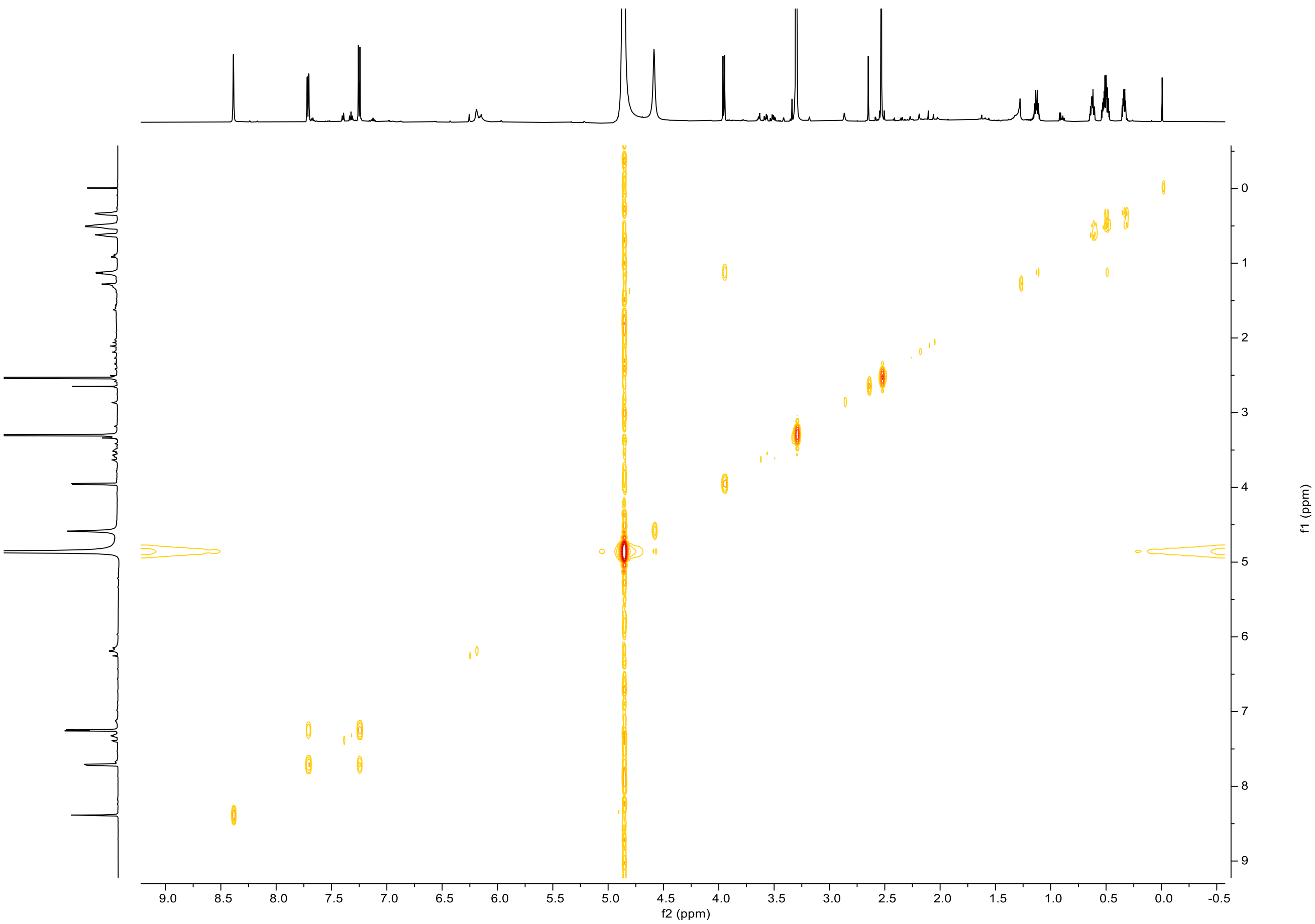

Figure S16: ^{1}H-^{1}H COSY spectrum of compound 2
